# Pinopsin evolved as the ancestral dim-light visual opsin in vertebrates

**DOI:** 10.1038/s42003-018-0164-x

**Published:** 2018-10-01

**Authors:** Keita Sato, Takahiro Yamashita, Keiichi Kojima, Kazumi Sakai, Yuki Matsutani, Masataka Yanagawa, Yumiko Yamano, Akimori Wada, Naoyuki Iwabe, Hideyo Ohuchi, Yoshinori Shichida

**Affiliations:** 1 Department of Cytology and Histology, Graduate School of Medicine, Dentistry and Pharmaceutical Sciences, Okayama University, Okayama, 700-8558 Japan; 2 Department of Biophysics, Graduate School of Science, Kyoto University, Kyoto, 606-8502 Japan; 3 Cellular Informatics Laboratory, RIKEN, Wako, 351-0198 Japan; 4 Department of Organic Chemistry for Life Science, Kobe Pharmaceutical University, Kobe, 658-8558 Japan; 5 Research Organization for Science and Technology, Ritsumeikan University, Kusatsu, Shiga 525-8577 Japan

## Abstract

Pinopsin is the opsin most closely related to vertebrate visual pigments on the phylogenetic tree. This opsin has been discovered among many vertebrates, except mammals and teleosts, and was thought to exclusively function in their brain for extraocular photoreception. Here, we show the possibility that pinopsin also contributes to scotopic vision in some vertebrate species. Pinopsin is distributed in the retina of non-teleost fishes and frogs, especially in their rod photoreceptor cells, in addition to their brain. Moreover, the retinal chromophore of pinopsin exhibits a thermal isomerization rate considerably lower than those of cone visual pigments, but comparable to that of rhodopsin. Therefore, pinopsin can function as a rhodopsin-like visual pigment in the retinas of these lower vertebrates. Since pinopsin diversified before the branching of rhodopsin on the phylogenetic tree, two-step adaptation to scotopic vision would have occurred through the independent acquisition of pinopsin and rhodopsin by the vertebrate lineage.

## Abstract (web summary)

Sato et al. show that pinopsin, an extraocular opsin, is also expressed in a subpopulation of retinal photoreceptor cells in lower vertebrates. Its retinal expression coupled to its low thermal isomerization rate suggests that pinopsin can function as a visual pigment and provides some insights into the evolution of scotopic vision in vertebrates.

## Introduction

Vertebrate vision consists of scotopic and photopic vision. Most vertebrates have two types of photoreceptor cells in their retinas, namely rods and cones, that serve as primary photo-sensors for scotopic and photopic vision, respectively^1^. Visual pigments function as photoreceptive molecules in vertebrate photoreceptor cells and belong to the opsin family. They activate transducin (Gt) in a light-dependent manner to drive the phototransduction cascade in these cells. Vertebrate visual pigments are classified into five groups, one rhodopsin (rod pigment) group and four cone pigment groups, based on their amino acid sequences^2,3^. Phylogenetic analysis of visual pigments provided a simple answer to the question about the ancestral visual pigment. Cone pigments diversified into four groups (red, green, blue, and violet/UV-sensitive groups) first and the rhodopsin group later branched from one of the cone pigment groups. The current model for the acquisition of color and dim-light vision was thereby proposed, which assumes that color vision under photopic conditions originated first and low light vision developed later in early vertebrate evolutionary history.

Vertebrates have been shown to possess a variety of opsin genes in addition to visual pigments, which are thought to be responsible for non-visual photoreception^4^. Pinopsin is the first opsin to be characterized in an extraocular organ. It was originally isolated from the chicken pineal gland and functions as a blue-sensitive photopigment^5^. In non-mammalian vertebrates, the pineal gland is a photoreceptive endocrine organ that synthesizes melatonin^6–8^. Thus, it has been suggested that pinopsin can regulate the production and secretion of melatonin from the chicken pineal gland^9^. After the discovery of chicken pinopsin, pinopsin genes were discovered among many vertebrates ranging from aves (birds), reptiles, and amphibians, but not among mammals and teleosts^10^. The opsin phylogenetic tree shows that pinopsin is the non-visual opsin most closely related to visual pigments (Supplementary Figure 1). This is supported by the existence of molecular properties common to both pinopsin and visual pigments. Upon absorbing a photon, pinopsin converts to MII intermediate, whose absorption maximum (*λ*_max_) lies in the UV region, to couple with Gt^11^. These properties are not observed in other closely related non-visual opsins, such as VA opsin and parapinopsin, found in vertebrates^12,13^. Thus, it has been proposed that although pinopsin has the potential to function as a visual pigment, its exclusive expression in the brain results in the contribution of pinopsin to extraocular photoreception rather than visual photoreception.

Recent genomic analysis of various non-teleost fishes has revealed that the pinopsin gene can be found in the genomes of spotted gar^14,15^, coelacanth^16^, and elephant shark^17^ (Supplementary Figure 1). In addition, we isolated pinopsin from gray bichir, Siberian sturgeon, and spotted African lungfish. In this study, we show that pinopsin is expressed in the eyes of cartilaginous fish, gar, sturgeon, bichir, lungfish, and anuran species in addition to their brains. Through the histochemical analysis, expression of pinopsin could be detected in a small population of the retinal photoreceptor cells of spotted gar and *Xenopus tropicalis*. Moreover, the retinal chromophore of pinopsins shows a low thermal isomerization rate similar to that of rhodopsin, which is important for functioning under dim-light conditions. These data suggest that fish and anuran pinopsin can function as a visual pigment in the retina for dim- light vision. We revisit the evolutionary position of pinopsin in the context of color vision and dim-light vision acquisition.

## Results

### Pinopsin expression in the eyes and brain of vertebrates

**Fig. 1:**
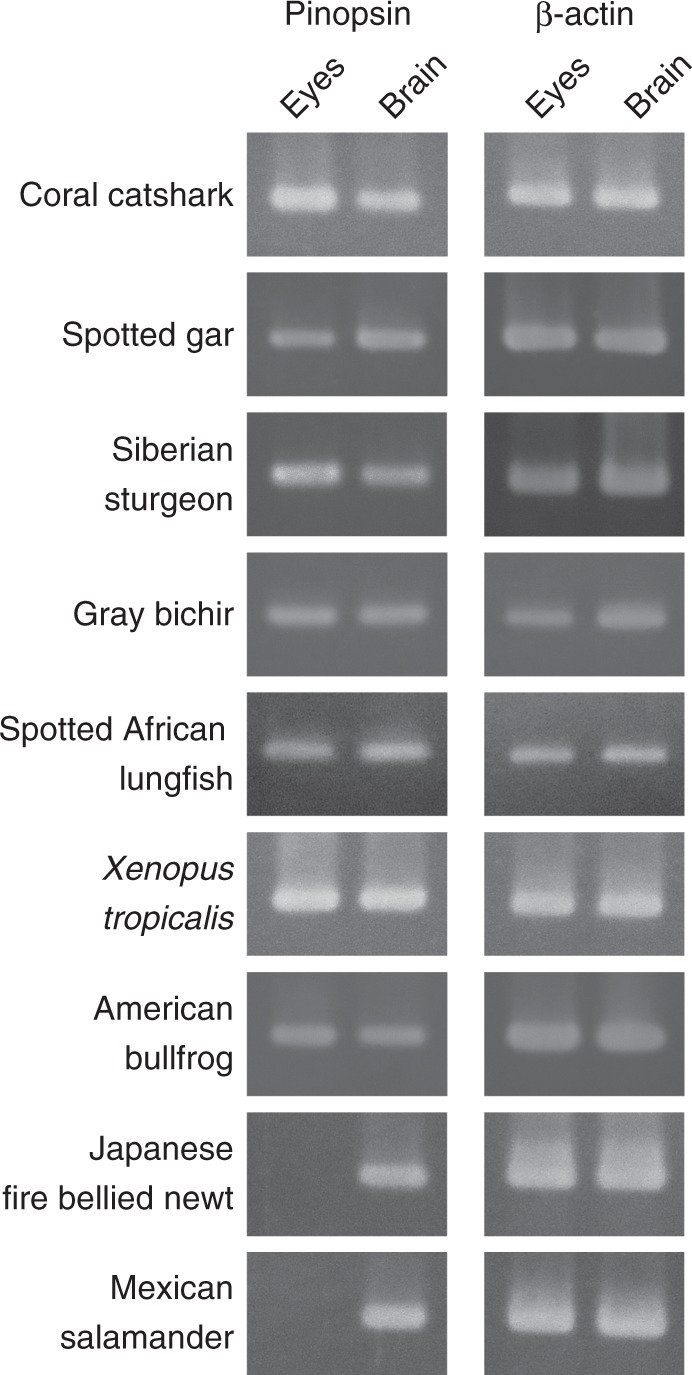
RT-PCR analysis of pinopsin expression in eyes and brains of non-teleost fishes and amphibians. Pinopsin transcript was detected from brains of all the fishes and amphibians investigated. The transcript was also detected from eyes of non-teleost fishes (coral catshark, spotted gar, Siberian sturgeon, gray bichir, and spotted African lungfish) and anurans (*X. tropicalis* and American bullfrog), but not from eyes of urodelans (Japanese fire bellied newt and Mexican salamander). β-actin transcript was detected from all the samples as an internal standard. Sequences of PCR primers and amplified sizes of each PCR are shown in Supplementary Table 1. Full gel images are shown in Supplementary Figure 2

Previous analyses of the distribution patterns of pinopsin in aves and reptiles have shown that pinopsin is exclusively expressed within the brain, especially in the pineal-related organs such as pineal gland and parietal eyes^5,18–21^. One exception is the diurnal gecko, *Phelsuma madagascariensis longinsulae*, whose pinopsin is expressed in a restricted population of cone cells in the pure-cone retina^22^. In this study, we first investigated whether pinopsin mRNA is expressed in the brain and eyes of a wide range of non-teleost fishes, namely coral catshark (*Atelomycterus marmoratus*) in the Chondrichthyes, spotted gar (*Lepisosteus oculatus*), Siberian sturgeon (*Acipenser baerii*), and gray bichir (*Polypterus senegalus*) in Class Actinopterygii, and spotted African lungfish (*Protopterus dolloi*) in Class Sarcopterygii (Fig. 1 and Supplementary Figure 2). Interestingly, RT-PCR analysis detected the expression of pinopsin mRNA not only in the brain but also in the eyes of these fish species. Pinopsin is therefore generally expressed in the eyes of non-teleost fishes. In addition, we analyzed mRNA expression of pinopsin in the eyes and brain of amphibians. Between the two urodelan species we examined, namely Japanese fire bellied newt (*Cynops pyrrhogaster*) and Mexican salamander (*Ambystoma mexicanum*), we detected pinopsin mRNA in the brain, but not in the eyes, by RT-PCR. However, between the two anuran species we examined, namely western clawed frog (*X. tropicalis*) and American bullfrog (*Rana catesbeiana*), pinopsin was expressed in both the eyes and brain. These results show that pinopsin is expressed in the eyes of non-teleost fishes and anurans.

### Pinopsin distribution pattern in vertebrate retinas

**Fig. 2:**
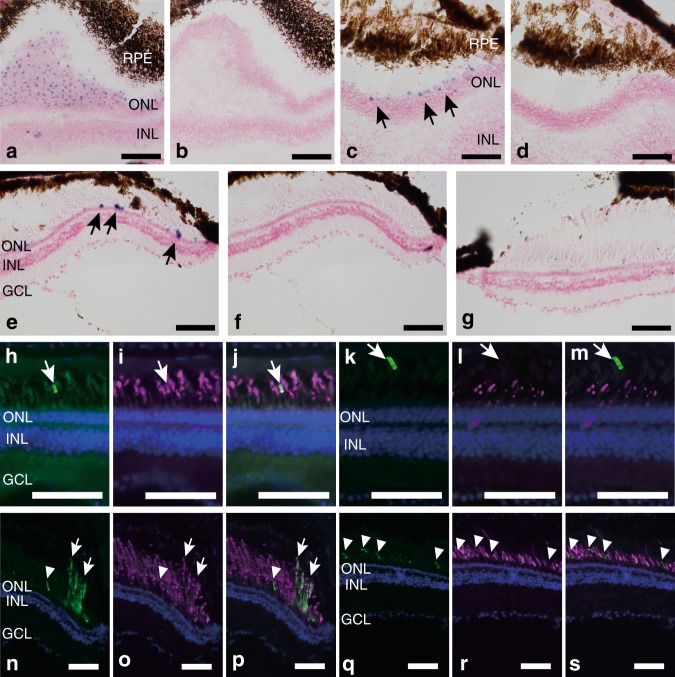
Distribution of pinopsin in the retina of spotted gar and *X. tropicalis.* **a**–**d** Detection of spotted gar pinopsin mRNA in the retina by in situ hybridization analysis. Horizontal (**a, b**) or frontal (**c, d**) consecutive sections were hybridized with pinopsin antisense (**a, c**) and sense (**b, d**) probes. Arrows indicate the positions of hybridization signals. **e**–**g** Detection of *X. tropicalis* pinopsin mRNA in the retina by in situ hybridization analysis. Frontal consecutive sections were hybridized with pinopsin antisense (**e, g**) and sense (**f**) probes. Dorsal region (**e, f**) and ventral region (**g**) of retina are shown, respectively. All the sections shown in panels **a**–**g** were counterstained with nuclear fast red. **h**–**j** Double immunofluorescence staining in the spotted gar retina showing pinopsin (**h**, green), rhodopsin (**l**, magenta), and the merge image (**j**). **k**–**m** Double immunofluorescence staining in the spotted gar retina showing pinopsin (**k**, green), red-sensitive cone pigment (**l**, magenta), and the merge image (**m**). White arrows indicate the positions of the positive signals of anti-pinopsin antibody. **n**–**p** Double immunofluorescence staining in the *X. tropicalis* retina showing pinopsin (n, green), rhodopsin (**o**, magenta), and merge images (**p**). **q**–s Double immunofluorescence staining in the *X. tropicalis* retina showing pinopsin (**q**, green), anti-red-sensitive cone pigment (r, magenta), and merge images (s). White arrows and arrow heads indicate the positions of the positive signals of anti-pinopsin antibody in outer segments of rod and cone photoreceptor cells, respectively. Nuclei in the sections shown in panels (**h**–s) were counterstained with Hoechst33342 (blue). Scale bar: **a**, **b**, 100 μm; **c**–**s**, 50 μm

To identify the detailed expression patterns of pinopsin, we investigated the distribution of pinopsin transcript in the retina and brain by in situ hybridization. The tissue distribution pattern of pinopsin mRNA was determined in the retina and brain of spotted gar and *X. tropicalis*. We observed abundant expression of pinopsin mRNA in the pineal gland of spotted gar and *X. tropicalis* (Supplementary Figure 3A–D), which is consistent with the results from aves and reptiles. We also successfully detected hybridization signals of pinopsin in the spotted gar retina (Fig. 2a–d).

We observed evidence of pinopsin transcript in the outer nuclear layer and photoreceptor layer at the dorsal edge of horizontal and frontal sections of the retina. In addition, in the *X. tropicalis* retina, hybridization signals of pinopsin were detected in the outer nuclear layer and photoreceptor layer on the dorsal side, but not on the ventral side (Fig. 2e–g).

To compare the expression patterns of pinopsin with those of visual pigments, we investigated the distribution of visual pigment transcripts in the retina of spotted gar and *X. tropicalis*. The spotted gar genome contains rhodopsin gene and five cone pigment genes that encode red-, green-, and blue-sensitive pigments and two UV light-sensitive pigments^23^ (Supplementary Figure 1). Expression of all these visual pigments was widely detected in the outer nuclear layer and photoreceptor layer (Supplementary Figure 3E–K). We confirmed that these visual pigments can form photopigments after reconstitution with the 11-cis form of A1 retinal (Supplementary Figure 4A–F). The distribution patterns of visual pigments in the retina contrast with the observation that the signals of pinopsin were only detected in a limited population of photoreceptor cells. *X. tropicalis* has rhodopsin gene and three cone pigment genes that encode red-, blue-, and violet-sensitive pigments (Supplementary Figure 1). It is well-known that blue-sensitive cone pigments are expressed in green rod cells which are unique to amphibians^24,25^. We also verified the expression of cone pigments in the *X. tropicalis* retina (Supplementary Figure 3L–N). As shown in other frog species, transcripts of red-sensitive cone pigments were abundantly detected in the outer nuclear layer and photoreceptor layer of the retina. The distributions of blue- and violet-sensitive cone pigments were found to be sparse in the outer nuclear layer and photoreceptor layer. The distribution density of pinopsin transcript hybridization signals was similar to that of the violet-sensitive cone pigment.

Furthermore, to determine whether pinopsin is expressed in rod or cone cells, and to investigate the subcellular distribution of pinopsin, retinas of spotted gar and *X. tropicalis* were stained with an antibody raised against the C-terminus of spotted gar and *X. tropicalis* pinopsin, respectively (Fig. 2h–s). The antibody against spotted gar pinopsin appeared to label the outer segments of a very small number of rod cells in the spotted gar retina. During the double staining with antibodies against rhodopsin and red-sensitive cone pigments, the positive signals of pinopsin overlapped with the staining by the antibody against rhodopsin, but not with those due to the antibody against the red-sensitive cone pigment in the spotted gar retina (Fig. 2h–m). In the *X. tropicalis* retina, the antibody against *Xenopus* pinopsin also labeled the outer segments of the localized small cluster of rod cells, which were merged with the immunoreactivity of the antibody against rhodopsin (Fig. 2n–p). In addition, we observed the expression of pinopsin in the shorter outer segments of a minor population of photoreceptor cells (Fig. 2q–s). These signals were not merged with the clear immunoreactive signals of rhodopsin or red-sensitive cone pigments and were quite similar to the staining patterns observed for the antibody against the red-sensitive cone pigment. In summary, these immunofluorescence staining data showed that pinopsin is co-expressed with rhodopsin in the outer segments of a very small population of rod cells in the spotted gar and *X*. *tropicalis* retina and that pinopsin is also localized in the outer segments of a small subset of non-red cone cells in the *X. tropicalis* retina. The ratios of pinopsin-positive cells were approximated to be 0.40, 0.51, and 1.9% in spotted gar rod cells, *X. tropicalis* rod cells, and *X. tropicalis* cone cells, respectively (Supplementary Figure 5).

### Molecular characteristics of pinopsin

To obtain insights into the functional aspect of pinopsin expression in retinal photoreceptor cells, we investigated the molecular properties of pinopsin in comparison with visual pigments. We prepared the recombinant proteins of spotted gar and *Xenopus* pinopsin after reconstitution with 11-cis form of A1 retinal. The absorption spectra of the purified proteins of spotted gar and *Xenopus* pinopsin exhibited a *λ*_max_ at 477 nm and 470 mm, respectively (Supplementary Figure 4G, H), which are quite similar to that of chicken pinopsin (468 nm)^5,11^.

**Fig. 3:**
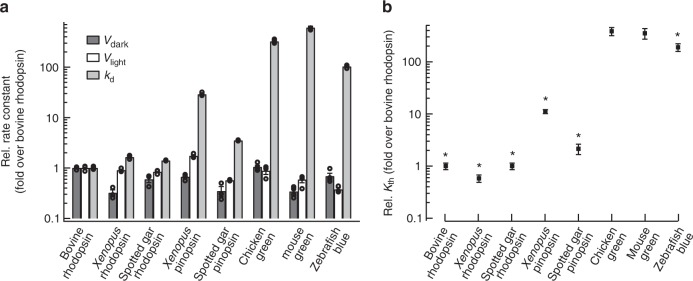
Comparison of the thermal activation rate between pinopsin and visual pigments. **a** The *v*_dark_, *v*_light_, and *k*_d_ of bovine rhodopsin, *X. tropicalis* rhodopsin, spotted gar rhodopsin, *X. tropicalis* pinopsin, spotted gar pinopsin, and three cone pigments (mouse green-sensitive one, chicken green-sensitive one, and zebrafish blue-sensitive one). The values of bovine rhodopsin, spotted gar rhodopsin, *X. tropicalis* pinopsin, and spotted gar pinopsin were calculated from the results shown in Supplementary Figure 6. The values of *X. tropicalis* rhodopsin and three cone pigments were referred to our previous studies^28,29^. **b** Comparison of *k*_th_ of rhodopsins, pinopsins, and cone pigments. The *k*_th_ values were estimated from data in panel **a** (see details in Methods). Error bars represent the standard error of the mean estimated based on more than three independent measurements. An asterisk (*) indicates a significant difference in relative rate constants between opsins and chicken green-sensitive cone pigment (*P* < 0.05; Student’s *t* test, two-tailed)

Pinopsin can activate Gt, which means it is possible that pinopsin may function as a visual pigment by triggering a Gt-mediated signal transduction cascade in retinal photoreceptor cells^11^. It has been revealed that the molecular properties of visual pigments regulates the visual sensitivity of animals^26,27^. That is, vertebrate rhodopsin in rod cells exhibits the low thermal isomerization rate of the retinal chromophore, which leads to the low threshold of light detection in animals. Our immunohistochemical analysis revealed that pinopsin is expressed in rod cells of both the spotted gar and *X. tropicalis* retinas. To function as a visual pigment in rod cells, pinopsin also requires a lower thermal isomerization rate than cone pigments. Thus, we compared the thermal isomerization rates of the retinal chromophore between pinopsin and visual pigments. Recently, we developed a biochemical assay method to measure the thermal isomerization rate of the retinal chromophore of visual pigments^28,29^. Thermal isomerization rates (*k*_th_) are calculated using three experimentally determined values, namely *v*_dark_, *v*_ligth_, and *k*_d_ (see details in Supplementary Figure 6A). We determined the *v*_dark_, *v*_light_, and *k*_d_ of rhodopsin and pinopsin from spotted gar and *X. tropicalis* (Supplementary Figure 6) and calculated their *k*_th_ (Fig. 3). Spotted gar and *Xenopus* rhodopsins exhibited thermal isomerization rates >200-fold lower than those of cone visual pigments. The thermal activation rates of pinopsin were >20-fold lower than those of cone visual pigments. In particular, the rate of spotted gar pinopsin was comparable to that of rhodopsin. This result showed that pinopsin exhibits a low thermal isomerization rate to be able to function as a rhodopsin-like visual pigment.

## Discussion

On the opsin phylogenetic tree, vertebrate visual pigments form an independent group together with vertebrate non-visual opsins, pinopsin, VA opsin, parapinopsin, and parietopsin. These four opsins were originally identified in extraocular tissues of non-mammalian vertebrates and have since been identified among various vertebrates, but not among invertebrates^30^. Among these, genes encoding VA opsin, parapinopsin, and parietopsin can be found in both Actinopterygii and Sarcopterygii, which suggests that the common ancestor of both classes had already acquired these opsin genes. By contrast, a pinopsin gene was identified from aves, reptiles, and amphibians but not from teleost genomes. Recent genomic analysis of non-teleost fishes has revealed the pinopsin gene in coelacanth, spotted gar, and cartilaginous fishes^15–17^. We also identified the pinopsin gene in bichir, sturgeon, and lungfish. Thus, the pinopsin gene has been preserved in the major lineages of non-teleost fishes. This suggests that the common ancestor of the gnathostomes had already acquired the pinopsin gene and that the gene was independently lost from the mammalian and teleost lineages. We speculate that teleosts instead utilized exo-rhodopsin as a pineal opsin^31^. Altogether, these four opsins, pinopsin, VA opsin, parapinopsin, and parietopsin would have emerged together with five vertebrate visual pigment groups in the early evolutionary history of vertebrates.

In the present study, we detected the expression of pinopsin mRNA in both eyes and brain of non-teleost fishes and frogs. Specifically, pinopsin is distributed in the retinal photoreceptor cells of spotted gar and *X. tropicalis*. Thus, we provide the possibility that the common ancestor of the gnathostomes would have expressed pinopsin in both the retina and pineal gland for visual and non-visual photoreception. Disappearance of the expression of pinopsin in the retina would have independently occurred in the lineages of amphibians and sauropsids. The pineal gland and retina are thought to have common developmental origins^32^. In particular, non-mammalian vertebrates have morphologically and functionally similar cell types, photoreceptor cells, and projection neurons, in these tissues^33^. It has been shown that common retinal/pineal- specific transcription factors such as *Otx2* and *Crx* work in these tissues^34,35^. In addition, we can find a pineal regulatory element, which is recognized by Crx, in the sequences of the 5′-flanking regions of elephant shark (*Callorhinchus milii*), spotted gar, and *Xenopus* pinopsin genes (Supplementary Figure 7)^36^. Detailed functional analysis of the 5′-flanking regions of various pinopsin genes will help identify *cis*-element(s) responsible for the silencing of pinopsin expression in the retina.

Absorption spectra of visual pigments in frog photoreceptor cells have been examined by the microspectrophotometry^37^. Red rods contain green-sensitive photopigments, which is accounted for by rhodopsin. Accessory cones also contain green-sensitive photopigments despite the absence of green-sensitive cone pigment in the frog genomes. Accessory cones could be labeled by the antibodies against red-sensitive cone pigments, not by the antibodies against rhodopsin^38^, which suggests the possibility that the cells contain pinopsin that is closely related with red-sensitive cone pigment in the phylogenetic tree. However, because of the distribution of pinopsin-positive cone cells and the blue-shifted *λ*_max_ of pinopsin, the microspectrophotometric measurement of the accessory cones cannot be accounted for by pinopsin. Thus, the presence of pinopsin-expressing rod or cone cells has never been suggested by the previous microspectrophotometrical or electrophysiological studies. This would be due to small population and localized distribution of pinopsin-expressing cells and lower abundance of pinopsin.

Our biochemical analysis of pinopsin showed that the thermal isomerization rate of the retinal in pinopsin was much lower than those of cone pigments and was comparable to that in rhodopsin. The thermal isomerization of retinal in rhodopsin results in the thermal activation of rhodopsin, which leads to the discrete noise of rod cells^28,39,40^. This discrete noise event is indistinguishable from true single photon response induced by photo-activation of rhodopsin^41,42^. Thus, the discrete noise sets a limit for the absolute visual threshold in dim-light vision^26,27^. Under the same framework, the low thermal isomerization of the retinal in pinopsin can allow the pigment to contribute to dim-light vision. We previously reported that the lower thermal isomerization rate of retinal in rhodopsin compared with those in cone pigments is attributable to two amino acid residues at positions 122 and 189 (in the bovine rhodopsin numbering system)^28^. The pinopsin group has conserved residues at these positions, Ile122 and Pro189. These residues are also conserved in the red-sensitive cone pigment group, but not in the rhodopsin group. Thus, the difference of the thermal isomerization rates between cone pigments and pinopsin may be explained by other amino acid residues. It has been reported that the active state of pinopsin decays more slowly than those of cone pigments, which can be accounted for by a loss of two amino acids in the second extracellular loop of pinopsin^43^. We previously revealed that there is a strong relationship between thermal isomerization rates of the retinal chromophore of visual pigments in the dark state and decay rates of the active state of the pigments^28,29^. Altogether, we speculate that a shortened second extracellular loop is also responsible for lower thermal isomerization rates of the retinal chromophore of pinopsin.

**Fig. 4:**
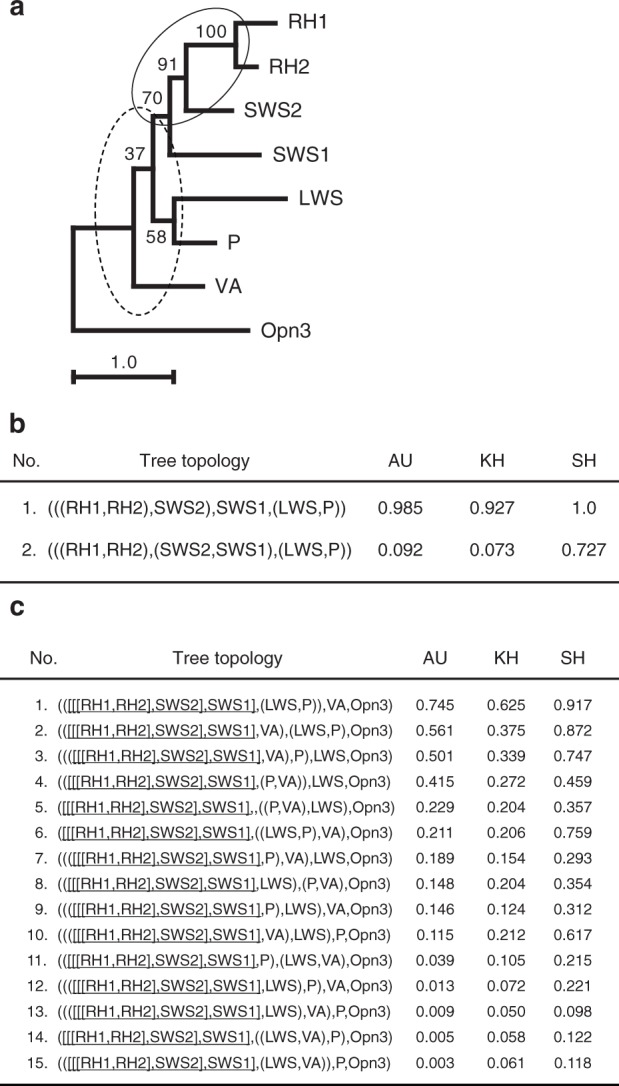
Detailed analysis of phylogenetic relationship between pinopsin and visual pigments. **a** Molecular phylogenetic tree of pinopsin and visual pigments inferred by maximum likelihood (ML) method. The ML tree was inferred by using JTT-tm model^53^ and Yang’s discrete gamma model^50^ with an optimized shape parameter alpha of 0.93. A sequence alignment of 249 amino acids in length was used for the tree inference. The numbers at each branch are bootstrap probabilities^51,54^. Partial tree topologies indicated by solid and broken ellipses were statistically tested in (**b**) and (**c**), respectively. RH1, RH2, SWS1, SWS2, LWS, P, VA, and Opn3 denote chicken rhodopsin, green-sensitive opsin, violet-sensitive opsin, blue-sensitive opsin, red-sensitive opsin (iodopsin), pinopsin, vertebrate ancient opsin, and Opsin3, respectively. **b** ML and other tree topologies of six opsins not statistically rejected by AU test. The ML tree of six opsins was inferred by the same method and models (alpha = 0.71) of (**a**). A highly reliable sequence alignment of 290 amino acids in length was used for the tree construction. Out of all the 105 tree topologies for six operational taxonomic units (OTUs), RH1, RH2, SWS1, SWS2, LWS, and P, the ML (tree number 1) and only one (tree number 2) topologies were not rejected by AU test^55^ at the 5% statistical significance level. The values of KH^54^ and SH^56^ statistical tests for these tree topologies are also represented in the list. **c** ML and other 14 tree topologies of eight opsins. Partial tree topology denoted by [[[RH1, RH2], SWS2], SWS1] was fixed in this analysis because the topology was contained in the ML tree of (**a**) and strongly supported by the statistical test of (**b**). Out of all the 15 tree topologies for five OTUs, [[[RH1, RH2], SWS2], SWS1], LWS, P, VA, and Opn3, the ML (tree number 1) and other nine (tree numbers 2 to 10) topologies were not rejected by AU test at the 5% level of significance. The values of KH and SH tests are also shown in the list

To analyze the detailed phylogenetic relationship between vertebrate visual pigments and pinopsin, we constructed a phylogenetic tree for vertebrate visual and non-visual opsins using the maximum likelihood method (Fig. 4 and Supplementary Figure 8). Although we presented the same maximum likelihood tree topology irrespective of opsin members in Fig. 4a and Supplementary Figure 8A, there were multiple possible topologies about the diversification of pinopsin, the red-sensitive cone pigment, and the ancestor of other cone pigments that were not statistically rejected as shown in Fig. 4c and Supplementary Figure 8C. Therefore, we could not determine if either pinopsin was branched before or after branching of the red-sensitive cone pigment and the ancestor of other cone pigments. This means that pinopsin, the red-sensitive cone pigment, and the ancestor of other cone pigments diversified by gene duplication around the same time. This phylogenetic analysis suggests that pinopsin branched from other opsins before the branching of rhodopsin and the green-sensitive cone pigment. Therefore, we can propose an evolutionary model for color and dim-light vision in which the vertebrate ancestor underwent adaptive process to scotopic vision twice. According to this model, pinopsin emerged during the early evolutionary history of color vision and rhodopsin originated after the evolution of color vision.

**Fig. 5:**
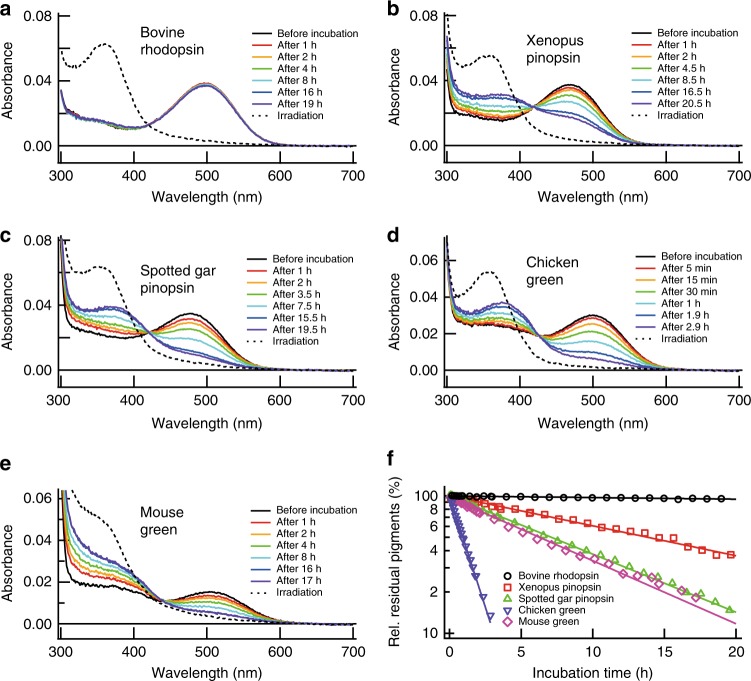
Comparison of thermal stability among rhodopsin, pinopsin, and cone pigments. Bovine rhodopsin (**a**), Xenopus pinopsin (**b**), spotted gar pinopsin (**c**), chicken green-sensitive cone pigment (**d**), and mouse green-sensitive cone pigment (**e**) after purified in 0.02% DM were incubated at 37 °C and spectral changes were measured. After the incubation in the dark, the samples were mixed with 50 mM hydroxylamine and irradiated with yellow light (> 480 nm) to estimate the amount of the pigments. Difference spectra were obtained by subtracting the spectrum before incubation at 37 °C from those measured after incubation and irradiation as shown in Supplementary Figure 9. **f** The amounts of residual pigments were plotted against incubation time at 37 °C. The amounts of residual pigments were estimated from the difference absorbance at 540 nm in Supplementary Figure 9

According to this evolutionary model, it is not apparent why pinopsin has hardly been used for dim-light vision in extant vertebrates. To address this question, we compared the thermal stability of rhodopsin and pinopsin (Fig. 5 and Supplementary Figure 9). A lower thermal stability would produce higher amounts of apo-proteins of the pigments. A previous study of tiger salamander cone cells showed that cone cells contain a substantial amount of apo-proteins of visual pigments, which leads to the high basal activity and elevates the threshold of light detection^44^. Our analysis indicated that pinopsin gradually decayed during incubation in the dark, whereas rhodopsin was quite stable under the same conditions (Fig. 5f). The lower thermal stability of pinopsin suggests the possibility of increasing the basal activity and decreasing the photosensitivity of photoreceptor cells. Thus, we speculated that the vertebrate ancestor acquired rhodopsin as a more thermally stable visual pigment, which is advantageous for working as the main player in dim-light vision.

In conclusion, we have demonstrated the possibility that pinopsin functions as a visual pigment for dim-light vision in non-teleost fishes and anurans. This enables us to revisit the currently accepted answer to the question of how color and dim-light vision evolved. We speculate that the vertebrate ancestor underwent adaptation to dim-light vision twice, first through the evolution of pinopsin during the early acquisition process of color vision and subsequently by the evolution of rhodopsin after the acquisition of color vision. During the diversification of vertebrates, rhodopsin was selected as the universal visual pigment for dim-light vision and the expression pattern of pinopsin was altered for it to function mainly in pineal photoreception.

## Methods

### Animals and ethics statement

*Xenopus tropicalis*, and spotted gar (*Lepisosteus oculatus*: ~7 cm) were obtained from Institute for Amphibian Biology (Hiroshima University) through the National Bio-Resource Project of the MEXT, Japan, and Nippon Aquarium Co., Ltd., Tokyo, Japan, respectively. Coral catshark (*Atelomycterus marmoratus*: ~30 cm), gray bichir (*Polypterus senegalus*: ~15 cm), Siberian sturgeon (*Acipenser baerii*: ~20 cm), spotted African lungfish (*Protopterus dolloi*: ~20 cm), Japanese fire bellied newt (*Cynops pyrrhogaster*) and Mexican salamander (*Ambystoma mexicanum*) were purchased from a local pet shop. They were euthanized by immobilization using MS222 and immediate decapitation, and dissected just after they were brought into our laboratory. The use of animals in these experiments was in accordance with guidelines established by the Ministry of Education, Culture, Sports, Science and Technology of Japan. The protocols in this paper were approved by the Animal Care and Use Committee of Kyoto University (permit number: H2622, H2718, and H2815).

### Isolation of cDNA encoding opsin and β-actin

The full-length cDNAs of spotted gar rhodopsin (XM_006630625), red-sensitive cone pigment (XM_006625291), green-sensitive cone pigment (XM_006628473), blue-sensitive cone pigment (XM_006625292), UV-sensitive cone pigment1 (XM_006633219), and pinopsin (XM_015367820) were isolated by PCR from the 1st strand cDNA from eyes. Primer sequences are as follows: 5′-ATGAATGGCACAGAGGGCCC-3′ (forward) and 5′-TGCAGGAGACACCTGACTGG-3′ (reverse) for rhodopsin, 5′-ATGACGGAGCACTGGGGGAG-3′ (forward) and 5′-CGCGGGAGAGACGGAGGAAT-3′ (reverse) for red-sensitive cone pigment, 5′-ATGAATGGCACAGAGGGTAA-3′ (forward) and 5′-GGCCGGGGACACCTGACTGG-3′ (reverse) for green-sensitive cone pigment, 5′-ATGCGTCAACTTCGGCCCGA-3′ (forward) and 5′-CGCGGGGGCCACCTGACTGC-3′ (reverse) for blue-sensitive cone pigment, 5′-ATGGCAGGCACAGAAGAATT-3′ (forward) and 5′-GCTGGGGCTGACCTGACTTG-3′ (reverse) for UV-sensitive cone pigment1, and 5′-ATGCCCATCCTGGTGAACTC-3′ (forward) and 5′-AGCAGGGGTGACCTTGCTGC-3′ (reverse) for pinopsin, respectively. The clone of spotted gar UV-sensitive cone pigment2 (LC328977) was obtained from eyes based on the homology to the sequence of spotted gar UV-sensitive cone pigment1 (XM_006633219). Primer sequences are as follows: 5′-ATGGCAGGCACAGAAGAATT-3′ (forward) and 5′-GCTGGGGCTGACCTGACTTG-3′ (reverse). The full-length cDNAs of *X. tropicalis* red-sensitive cone pigment (BC135755), blue-sensitive cone pigment (XM_002937226), violet-sensitive cone pigment (BC166308), and pinopsin (XM_002934345) were isolated from eyes or brain. Primer sequences are as follows: 5′-ATGGCTTCCCATTGGAATGA-3′ (forward) and 5′-TGCAGGAGACACTGAAGAGT-3′ (reverse) for red-sensitive cone pigment, 5′-ATGAGTAAAGGCCGTGCAGA-3′ (forward) and 5′-TGACGGGGCAACCTGGCTGC-3′ (reverse) for blue-sensitive cone pigment, 5′-ATGAGAATGTTGGAAGAGGA-3′ (forward) and 5′-GGCAGGGCTGACTTGGCTGG-3′ (reverse) for violet-sensitive cone pigment, and 5′-ATGCGTGCTGGAAACATGTC-3′ (forward) and 5′-TGCAGGTGTGACCTTATTTC-3′ (reverse) for pinopsin, respectively. To isolate the clone of coral catshark pinopsin, we first obtained the sequences of exon 1 (including start codon of ORF) and exon 4 by PCR from genomic DNA based on the homology to the sequences of elephant shark (*Callorhinchus milii*) and whale shark (*Rhincodon typus*). Primer sequences are as follows: 5′-CCTGATTAATGAAACCACTTT-3′ (forward) and 5′-ACCTGTCAGAGACACCATGAAACC-3′ (reverse) for exon 1 and 5′-GTTGCTCAGCAACAGAAAGA-3′ (forward) and 5′-CTGTTTGTTCATGAAGACATAGAT-3′ (reverse) for exon 4. After identification of start codon, the partial ORF sequence of coral catshark pinopsin (LC328553) was obtained from eyes. Primer sequences are as follows: 5′-ATGGGTTTTTCCACGGCCCC-3′ (forward) and 5′-AATGGGATTGTAGACAGTGGC-3′ (reverse). The clone of coral catshark β-actin (LC328559) was obtained from eyes based on the homology to the sequences of elephant shark and whale shark. Primer sequences are as follows: 5′-CTTGTTGTTGACAATGGATC-3′ (forward) and 5′-CCATGCCAATTCATCTCGCG-3′ (reverse). To isolate the clone of Siberian sturgeon pinopsin, we first searched it in the transcriptome data deposited at the NCBI Sequence Read Archive (BioProject accession ID: PRJNA274436). We successfully identified multiple sequence reads corresponding to pinopsin and isolated the full-length ORF sequence of Siberian sturgeon pinopsin (LC365918) from brain. Primer sequences are as follows: 5′-ATGCAGAGCAATGACAGTAC-3′ (forward) and 5′-TTAGGCAGGGGTGACCTTGC-3′ (reverse). To isolate the clone of gray bichir pinopsin, we first searched it in the transcriptome data deposited at the NCBI Sequence Read Archive (BioProject accession ID: PRJNA269317). We successfully identified multiple sequence reads corresponding to pinopsin and isolated the partial ORF sequence of gray bichir pinopsin (LC328554) from brain. Primer sequences are as follows: 5′-CAGTGGCCCTACCAAGCACC-3′ (forward) and 5′-TTAGGCTGGGCTGACTTTGG-3′ (reverse). The clone of gray bichir β-actin (LC328560) was obtained from eyes based on the homology to the sequences of elephant shark, whale shark, spotted gar and coelacanth. Primer sequences are as follows: 5′-ATGGAAGATGAAATTGCCGC-3′ (forward) and 5′-TTAGAAGCATTTGCGGTGGA-3′ (reverse). To isolate the clone of spotted African lungfish, we first searched it in the transcriptome data of West African lungfish (*Protopterus annectens*) deposited at the NCBI Sequence Read Archive (BioProject accession ID: PRJNA282925)^45^. We successfully identified multiple sequence reads corresponding to pinopsin and isolated the full-length ORF sequence of spotted African lungfish pinopsin (LC328555) from brain. Primer sequences are as follows: 5′-TTGGGATTAACATTTCCAAG-3′ (forward) and 5′-ATTAGGTAAAGCTATGAGGC-3′ (reverse). The clone of spotted African lungfish β-actin (LC328561) was obtained from eyes based on the homology to the sequences of elephant shark, whale shark, spotted gar and coelacanth. Primer sequences are as follows: 5′-ATGGAAGATGAAATTGCCGC-3′ (forward) and 5′-TTAGAAGCATTTGCGGTGGA-3′ (reverse). To isolate the clone of American bullfrog pinopsin, we first obtained the sequences of exon 1 (including start codon of ORF) and exon 5 from genomic DNA based on the homology to the sequences of other frog pinopsins. Primer sequences are as follows: 5′-GATCCTCTTTGGAACTTTAAATCTC-3′ (forward) and 5′-GAAGCCTTCAAATTCACACA-3′ (reverse) for exon 1 and 5′-TTCCGAAACTGCTTAATGAC-3′ (forward) and 5′-TTATGCTGGTGTGACCTTGT-3′ (reverse) for exon 5. Based on the obtained sequence of exon 5, we performed 3′ rapid amplification of cDNA ends by 3′-Full RACE Core Set (Takara, Japan) to confirm the position of the stop codon of ORF. Gene-specific primer 5′-TTCCGAAACTGCTTAATGAC-3′ and adapter primer 5′-CTGATCTAGAGGTACCGGATCC-3′ were used. Finally, the full-length ORF sequence of American bullfrog pinopsin (LC328556) was obtained from eyes. Primer sequences are as follows: 5′-ATGAGGGCAGCGAATATGTC-3′ (forward) and 5′-TGCTGGTGTGACCTTGTTTC-3′ (reverse). To isolate the clone of Japanese fire bellied newt pinopsin, we first searched it in the database IMORI (the sequence resource for the Japanese fire bellied newt; http://antler.is.utsunomiya-u.ac.jp/imori/). We successfully obtained the clone including ORF sequence of pinopsin. The full-length cDNA of Japanese fire bellied newt pinopsin (LC328557) was isolated from brain. Primer sequences are as follows: 5′-ATGGAAAATGGCACCCCCGG-3′ (forward) and 5′-CGCTGGCGTGACCTTATTCC-3′ (reverse). To isolate the clone of Mexican salamander pinopsin, we first searched it in the database Sal-Site (Salamander Genome Project; http://www.ambystoma.org/). We successfully obtained the clone including partial ORF sequence of pinopsin. The partial cDNA of Mexican salamander pinopsin (LC328558) was isolated from brain. Primer sequences are as follows: 5′-GAAGCATGCTGTGATGGGCTG-3′ (forward) and 5′-TTCATGAAGACGTAAATAACG-3′ (reverse).

### RT-PCR analysis

Total RNA was isolated from eyes and brain including the pineal gland of coral catshark, spotted gar, Siberian sturgeon, gray bichir, spotted African lungfish, *X. tropicalis*, American bullfrog, Japanese fire bellied newt, and Mexican salamander. Reverse transcription was performed using oligo dT primer and PrimeScript II reverse transcriptase (Takara). PCR was performed for 35 cycles with gene-specific primers (Supplementary Table 1) using Expand High Fidelity DNA polymerase (SIGMA). Primers for pinopsin were designed on separate exons based on the sequences of coral catshark (LC328553), spotted gar (XM_015367820), Siberian sturgeon (LC365918), gray bichir (LC328554), spotted African lungfish (LC328555), *X. tropicalis* (XM_002934345), American bullfrog (LC328556), Japanese fire bellied newt (LC328557), and Mexican salamander (LC328558) clones. Primers for β-actin were designed on separate exons based on the sequences of coral catshark (LC328559), spotted gar (XM_006637121), Siberian sturgeon (JX027376), gray bichir (LC328560), spotted African lungfish (LC328561), *X. tropicalis* (BC068217), American bullfrog (AB094353), Japanese fire bellied newt (AB710471), and Mexican salamander (AY028791) clones. Each PCR reaction mixture was analyzed on agarose-TAE gel electrophoresis to visualize an amplified band under UV light after staining with ethidium bromide. Specific amplification of the target sequence was also confirmed using ABI3130xl genetic analyzer.

### Tissue sample preparation

After eyes and brains were dissected from *X. tropicalis* or spotted gar, they were fixed overnight in PBS-buffered 4% (*X. tropicalis*) or 6% (spotted gar) PFA at 4 °C for in situ hybridization, or fixed 30 min in PBS-buffered 4% PFA at 4 °C. Tissues were subsequently immersed in 20% sucrose in PBS overnight for cryoprotection and were frozen in a deep freezer in OCT compound (tissue tech). Frozen tissues were sliced into 16 μm sections and were attached to glass slides (MAS-GP typeA coated glass slide, Matsunami Glass Co., Ltd.), as previously described.^46^ Slides were stored in a dry chamber at −20 °C until use.

### In situ hybridization

In situ hybridization on tissue sections was performed as described previously^46^. In brief, digoxigenin-labeled RNA probes were synthesized from the cDNAs inserted into pBluescript II KS(+) or pTA2 (TOYOBO). All the following procedures were carried out at room temperature unless otherwise noted. Tissue sections were successively immersed in PBS-buffered 4% PFA for 15 min, methanol for 30 min, PBS for 5 min, Tris buffer (50 mM Tris-HCl, 5 mM EDTA, pH 7.6) containing 0.5 μg ml^−1^ proteinase K for 15 min, PBS for 5 min, PBS-buffered 4% PFA for 15 min, DEPC-treated water for 2 min, acetylation buffer (0.27% (v/v) acetic anhydride, 0.1 M triethanolamine, pH 8.0) for >10 min, PBS for 5 min, and hybridization buffer (0.75 M NaCl, 75 mM sodium citrate, 0.2 mg mL^−1^ yeast tRNA, 0.1 mg/mL heparin sodium, 1x Denhardt’s solution, 0.1% (v/v) Tween, 0.1% (w/v) CHAPS, 5 mM EDTA, 50% (v/v) formamide) at 65 °C for 3 h. After that, digoxigenin-labeled RNA sense or antisense probe diluted with hybridization buffer (final concentration: 0.17 μg mL^−1^) were applied to the specimens and were incubated for about 40 h at 65 °C. After hybridization, they were successively washed in SSC buffer (0.15 M NaCl, 15 mM sodium citrate, pH 7.0) containing 50% formamide for 15 min and for 1 h at 65 °C, one-fifth diluted SSC buffer for 1 h at 65 °C, and MABT (100 mM maleate, 150 mM NaCl, 0.1% Tween 20, pH 7.5) for three times 30 min. After washing, the sections were incubated with blocking buffer (1% (w/v) bovine serum albumin, 10% (v/v) sheep normal serum and 0.08% (v/v) Triton-X100 in PBS) for 30 min and were incubated with anti-digoxigenin Fab fragment conjugated with alkaline phosphatase (diluted 1:2000; Roche 11093274910) overnight at 4 °C. The slides were subsequently rinsed three times with MABT for 30 min, and twice with AP reaction buffer (100 mM Tris-HCl, 50 mM MgCl_2_, 100 mM NaCl, 0.1% Tween 20, pH 9.5) for 5 min. Finally, color development was performed with AP reaction buffer containing 5% (w/v) polyvinyl alcohol, 50 μg ml^−1^ nitroblue tetrazolium and 175 μg ml^−1^ 5-bromo-4-chloro-3-indolyl-phosphate.

### Immunohistochemistry

Specific antibody was raised to the C-terminus of spotted gar pinopsin or Xenopus pinopsin. Polyclonal antibody was commercially produced against a 16-amino acid (CDSSVTGTSTAGKTEI from spotted gar pinopsin or CRTDVTSVSEAGGNKV from Xenopus pinopsin) synthetic peptide conjugated to keyhole limpet hemocyanin by Protein Purify Ltd. (Gunma, Japan), according to their standard procedures. The antibodies were affinity purified prior to use by Protein Purify Ltd. By western blotting, we confirmed that the antibody recognized the recombinant proteins of spotted gar pinopsin or Xenopus pinopsin expressed in HEK293S cells (Supplementary Figure 10). Monoclonal anti-rhodopsin antibody, clone 4D2 (anti-rhodopsin; Millipore, MABN15) and anti-opsin antibody, Red/Green (anti-R/G; Millipore, AB5405) were also used as primary antibodies in this study. The secondary antibodies used for immunostaining in this study were Alexa Fluor 488 anti-guinea pig IgG (Invitrogen, A11073), Alexa Fluor 546 anti-rabbit IgG (Invitrogen, A11035), and Alexa Fluor 568 anti-mouse IgG (Invitrogen, A11031). Fluorescent immunostaining was performed by standard technique as follows. First, all sections were rehydrated by immersing for 5 min in PBS containing 0.1% Triton X-100 (PBSTx). They were blocked for 30 min at room temperature in PBSTx with 1% bovine serum albumin (blocking solution). All the antibodies were diluted in blocking solution. The sections were incubated with primary antibodies (anti-spotted gar pinopsin (diluted 1:500), anti-Xenopus pinopsin (diluted 1:2000), anti-rhodopsin (diluted 1:4000 for spotted gar retina, 1:2000 for Xenopus retina), and anti-R/G (diluted 1:500 for spotted gar retina, 1:2000 for Xenopus retina)) overnight at room temperature. Secondary antibodies were diluted to 1:1000 and incubated on the sections for 1 h at room temperature. Wash steps were performed four times with PBSTx for 5 min each after incubation with the primary and secondary antibodies. The cell nuclei were stained with 1 μg ml^−1^ Hoechst33342 included with the solution of secondary antibodies. The slides were mounted with home-made aqueous mounting media consisting of glycerol and polyvinyl alcohol.

### Preparation of recombinant proteins

The full-length cDNAs encoding opsins tagged with the epitope sequence of the anti-bovine rhodopsin monoclonal antibody Rho1D4 (ETSQVAPA) at the C-terminus were introduced into the mammalian expression vector, pCAGGS or pMT4, as described previously^46^. The plasmid DNA was transfected into the HEK293S cell line using the calcium phosphate method. The cell membranes were regenerated with 11-*cis* form of A1 retinal. The pigments were extracted, as previously described^29^, with Buffer A (50 mM HEPES, 140 mM NaCl, pH 6.5) containing 1% dodecyl maltoside (DM) and purified using Rho1D4-conjugated agarose. The purified pigments were eluted with 0.02% DM in Buffer A containing the synthetic C-terminal peptide of bovine rhodopsin. Absorption spectra of the pigments were measured to estimate their *λ*_max_. Sample preparation of the pigments for the measurements of *v*_dark_, *v*_light_, and *k*_d_ were performed as follows. The cell membranes expressing the pigments were divided into two aliquots. One was regenerated with 11-*cis*-retinal and 7-membered-ring 11-*cis*-retinal (7mr), and the other was regenerated by only 7mr. After regeneration, they were solubilized with Buffer A containing 1% DM and purified using Rho1D4-conjugated agarose. All the experiments after reconstitution of the pigments with retinal were performed under infrared light. We refer to the purified samples regenerated by both 11-*cis*-retinal and 7mr, or only 7mr as ‘pigment name-n’ or ‘pigment name-7mr’, respectively. We verified that the concentrations of the pigments contained in the two samples were similar by western blot analysis (Supplementary Figure 6).

### Spectrophotometry

Absorption spectra were recorded using a Shimadzu UV2450 spectrophotometer and an optical cell (width, 2 mm; light path, 1 cm). An optical cell-holder was connected to a Neslab RTE-7 temperature controller, which kept the sample temperature at 0 ± 0.1 °C (Supplementary Figure 4) or 37 ± 0.1 °C (Supplementary Figure 9).

### Estimation of thermal isomerization rate of retinal (*k*_th_)

The thermal isomerization rate of the retinal of the pigment was calculated using three experimentally determined values as previously described^28,29^. In brief, we assumed a simplified two-step reaction as shown in Supplementary Figure 6A, where R is the pigment in the inactive state and R* is the activated pigment by the thermal isomerization of the retinal. According to our previous reports^28^, the thermal isomerization rates (*k*_th_) are calculated from the following equation by using three experimentally determined values:

$$k_{{\mathrm{th}}} = \frac{{v_{{\mathrm{dark}}}}}{{v_{{\mathrm{light}}}}}k_{\mathrm{d}}$$

where *v*_dark_ is the initial rate of G protein activation by a pigment in the dark, *v*_light_ is the initial rate of G protein activation by an activated pigment, and *k*_d_ is the spontaneous decay rate of the activated pigment. The *v*_dark_ was measured by a [^35^S]GTPγS binding assay in the dark under infrared light. The assay mixture consisted of 300 nM pigments, 1 μM Gt, 5 μM GTPγS, 25 nM [^35^S]GTPγS, 0.015% DM, 50 mM HEPES (pH 6.5), 140 mM NaCl, 5.8 mM MgCl_2_, and 1 mM DTT. Experimental data were fitted by a single exponential function, and the *v*_dark_ was estimated by the difference between the initial rates between two samples (‘pigment name-n’ and ‘pigment name-7mr’). Estimation of the *v*_light_ was performed by measuring fluorescence emission changes of intrinsic tryptophan of Gt after flash irradiation. The assay mixture consisted of 20 nM pigments, 0 or 1 μM Gt, 5 μM GTPγS, 0.015% DDM, 50 mM HEPES (pH 6.5), 140 mM NaCl, 5.8 mM MgCl_2_, and 1 mM DTT. Experimental data were fitted by a single exponential function, and the *v*_light_ was estimated from the difference of initial rates of light-dependent fluorescence increase of pigment-n sample with and without Gt. Estimation of the *k*_d_ was carried out by measuring fluorescence emission changes of intrinsic tryptophan of pigment associated with retinal release. The assay mixture consisted of 20 nM pigments, 5 μM GTPγS, 0.015% DDM, 50 mM HEPES (pH 6.5), 140 mM NaCl, 5.8 mM MgCl_2_, and 1 mM DTT. Experimental data were fitted by a single exponential function to estimate *k*_d_. The mean and error of *k*_th_ in Fig. 3b were estimated based on the propagation of errors as follows:

$$M_{{\mathrm{th}}} = \frac{{M_{{\mathrm{dark}}}}}{{M_{{\mathrm{light}}}}}M_{\mathrm{d}}$$

$$\varepsilon _{{\mathrm{th}}} = \frac{{M_{{\mathrm{dark}}}}}{{M_{{\mathrm{light}}}}}M_{\mathrm{d}}\sqrt {\left( {\frac{{\varepsilon _{{\mathrm{dark}}}}}{{M_{{\mathrm{dark}}}}}} \right)^2 + \left( {\frac{{\varepsilon _{{\mathrm{light}}}}}{{M_{{\mathrm{light}}}}}} \right)^2 + \left( {\frac{{\varepsilon _{\mathrm{d}}}}{{M_{\mathrm{d}}}}} \right)^2}$$

where *Μ*_th_, *Μ*_dark_, *Μ*_light_, *Μ*_d_ were mean, and *ε*_th_, *ε*_dark_, *ε*_light_, *ε*_d_ were SEM of *k*_th_, *v*_dark_, *v*_light_, *k*_d_, respectively.

### Molocular phylogenetic analyses

The amino acid sequences of opsins were aligned by MAFFT^47^, and then manually inspected and improved, if necessary. Positions with alignment gaps and ambiguous alignment regions of weak sequence similarity were excluded from the phylogenetic analyses. The molecular phylogenetic tree of opsins (Supplementary Figure 1) was constructed by neighbor-joining (NJ) method^48^ using maximum likelihood (ML) distances calculated with JTT model^49^ and Yang’s discrete gamma model^50^ with an optimized shape parameter alpha. Bootstrap probabilities for the tree branches of the NJ tree were obtained by 1000 bootstrap resamplings^51^. The ML trees of pinopsin and other visual pigments (Fig. 4 and Supplementary Figure 8) were inferred by RAxML version 8^52^, a ML tree search program, using JTT-tm model^53^ and Yang’s discrete gamma model^50^ with an optimized shape parameter alpha. Bootstrap probabilities for the tree branches of the ML trees (Fig. 4a and Supplementary Figure 8A) were obtained by 200 bootstrap resamplings^51,54^. Statistical significance for ML and other possible tree topologies were evaluated by using approximately unbiased (AU)^55^, Kishino-Hasegawa (KH)^54^, and Shimodaira-Hasegawa (SH)^56^ tests. Accession numbers of the amino acid sequences of pinopsins and other visual pigments are as follows: chicken pinopsin, U15762; green iguana pinopsin, AB626971; schlegel’s Japanese gecko pinopsin, XM_015421659; Chinese softshell turtle pinopsin, XM_006138728; Chinese alligator pinopsin, XM_006016114; *X. tropicalis* pinopsin, XM_002934345; Japanese toad pinopsin, AF200433; American bullfrog pinopsin, LC328556; Japanese fire bellied newt pinopsin, LC328557; coelacanth pinopsin, XM_005987543; spotted gar pinopsin, XM_015367820; elephant shark pinopsin, XM_007896544; whale shark pinopsin, XM_020522554; coral catshark pinopsin, LC328553; human red, Z68193; human green, K03494; chicken red, X57490; *X. tropicalis* red, BC135755; spotted gar red, XM_006625291; human blue, U53874; chicken violet, M92039; *X. tropicalis* violet, BC166308; spotted gar UV1, XM_006633219; spotted gar UV2, LC328977; chicken blue, M92037; *X. tropicalis* blue, XM_002937226; spotted gar blue, XM_006625292; chicken green, M92038; spotted gar green, XM_006628473; human rhodopsin, U49742; chicken rhodopsin, D00702; *X. tropicalis* rhodopsin, BC135234; spotted gar rhodopsin, XM_006630625; spotted gar exo-rhodopsin, XM_006630940; chicken VA opsin, EF055883; spotted gar VA opsin, XP_006630456; sea lamprey (*Petromyzon marinus*) P-opsin, AAC41240; chicken Opn3, AB436160; spotted gar Opn3, XP_006638720; chicken TMT opsin, XP_004938575; spotted gar TMT opsin, XP_015219896; western honey bee (*Apis mellifera*) pteropsin, NP_001035057.

### Western blotting

Extracts from opsin-transfected or mock-transfected HEK293S cells were subjected to SDS-PAGE, transferred onto a polyvinylidene difluoride membrane, and probed with Rho1D4 (Supplementary Figure 6) or anti-pinopsin antibodies (Supplementary Figure 10). Dilutions of primary antibodies were 1:1000 for anti-spotted gar pinopsin and 1:1000 for anti-Xenopus pinopsin, respectively. For immunodetection by Rho1D4, undiluted culture supernatant of Rho1D4 hybridoma was used. The secondary antibodies used for western blotting in this study were horseradish peroxidase (HRP) conjugated anti-mouse IgG (Invitrogen, 626520) and HRP conjugated anti-guinea pig IgG (Invitrogen, 614620). Dilutions of secondary antibodies were 1:10,000 for HRP conjugated anti-mouse IgG and 1:2000 for HRP conjugated anti-guinea pig IgG, respectively. Immunoreactive proteins were detected by ECL (GE Healthcare) and visualized by a luminescent image analyzer (LAS 4000mini, GE Healthcare).

## Electronic supplementary material

supplementary information

## Data Availability

The datasets of the current study are available in the Dryad repository (10.5061/dryad.6np02kb)^57^. The sequences reported in this paper have been deposited in the GenBank database (accession nos. LC328553~LC328561, LC328977, and LC365918).
