# Supplementary material for: Pinopsin evolved as the ancestral dim-light visual opsin in vertebrates
Source: Commun Biol. 2018 Oct 1;1:156. doi: 10.1038/s42003-018-0164-x (PMC6167363; doi:10.1038/s42003-018-0164-x)
Supplement: Supplementary file 1 — supplementary information [file 42003_2018_164_MOESM1_ESM.pdf]

Supplementary Information for

Pinopsin evolved as the ancestral dim-light visual opsin in vertebrates

Keita Sato[a], Takahiro Yamashita[b,1], Keiichi Kojima[b], Kazumi Sakai[b] ,Yuki Matsutani[b], Masataka Yanagawa[c], Yumiko Yamano[d], Akimori Wada[d], Naoyuki Iwabe[b], Hideyo Ohuchi[a], Yoshinori Shichida[b,e,1]

[a]Department of Cytology and Histology, Graduate School of Medicine, Dentistry and Pharmaceutical Sciences, Okayama University, Okayama, Japan. [b]Department of Biophysics, Graduate School of Science, Kyoto University, Kyoto, Japan. [c]Cellular Informatics Laboratory, RIKEN, 2-1 Hirosawa, Wako 351-0198, Japan. [d]Department of Organic Chemistry for Life Science, Kobe Pharmaceutical University, Kobe 658-8558, Japan. [e]Research Organization for Science and technology, Ritsumeikan University, Kusatsu, Shiga 525-8577, Japan.

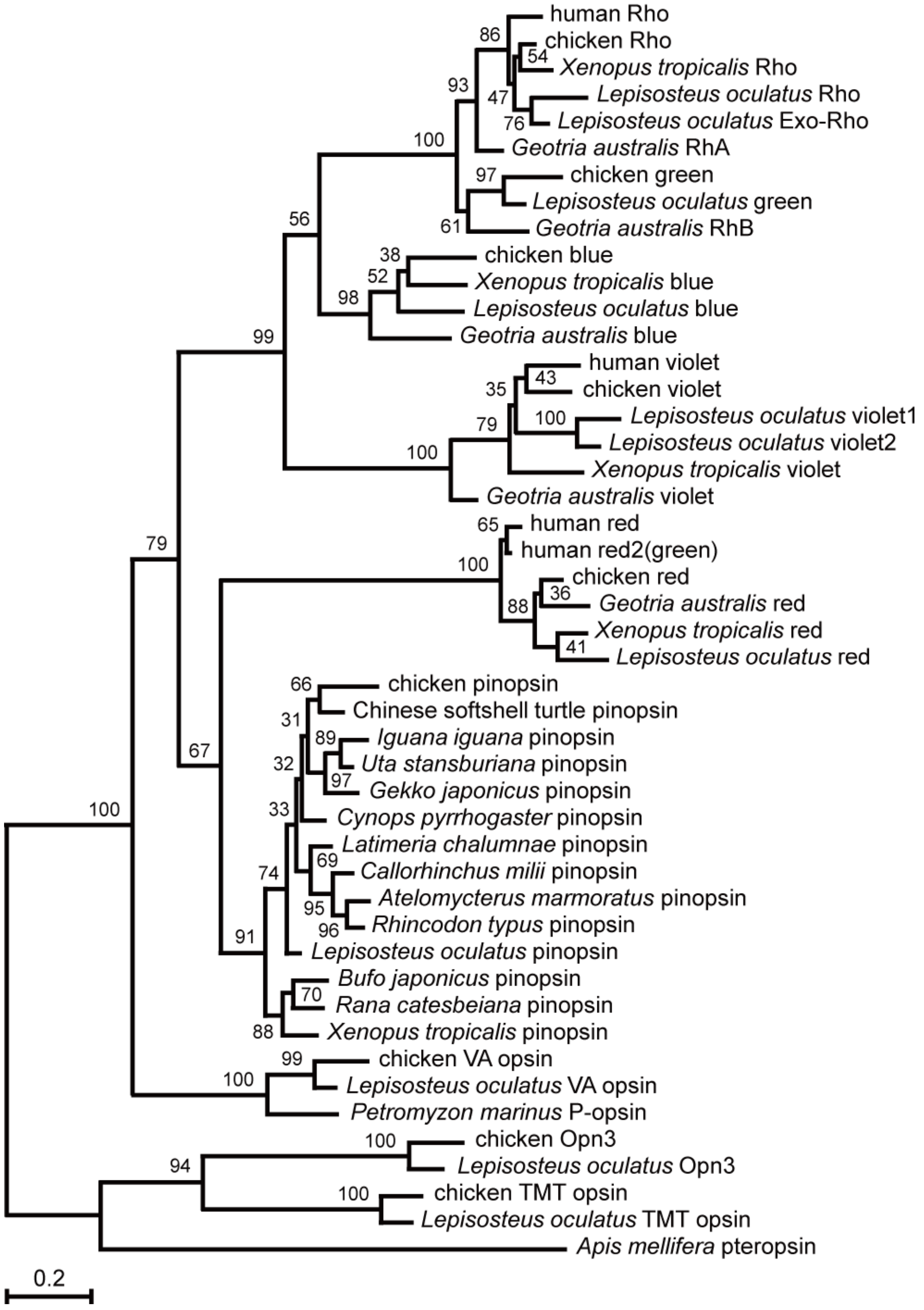

Supplementary Figure 1 Phylogenetic tree of pinopsin and visual pigments

The phylogenetic tree was constructed by neighbor joining method[1] using maximum likelihood distances calculated with JTT model[2] and Yang's discrete gamma model[3] with an optimized shape parameter alpha of 0.94. An unambiguous sequence alignment of 235 amino acids in length excluding gaps was used for the tree inference. The numbers at each tree branch are bootstrap probabilities obtain by 1,000 bootstrap resamplings[4].

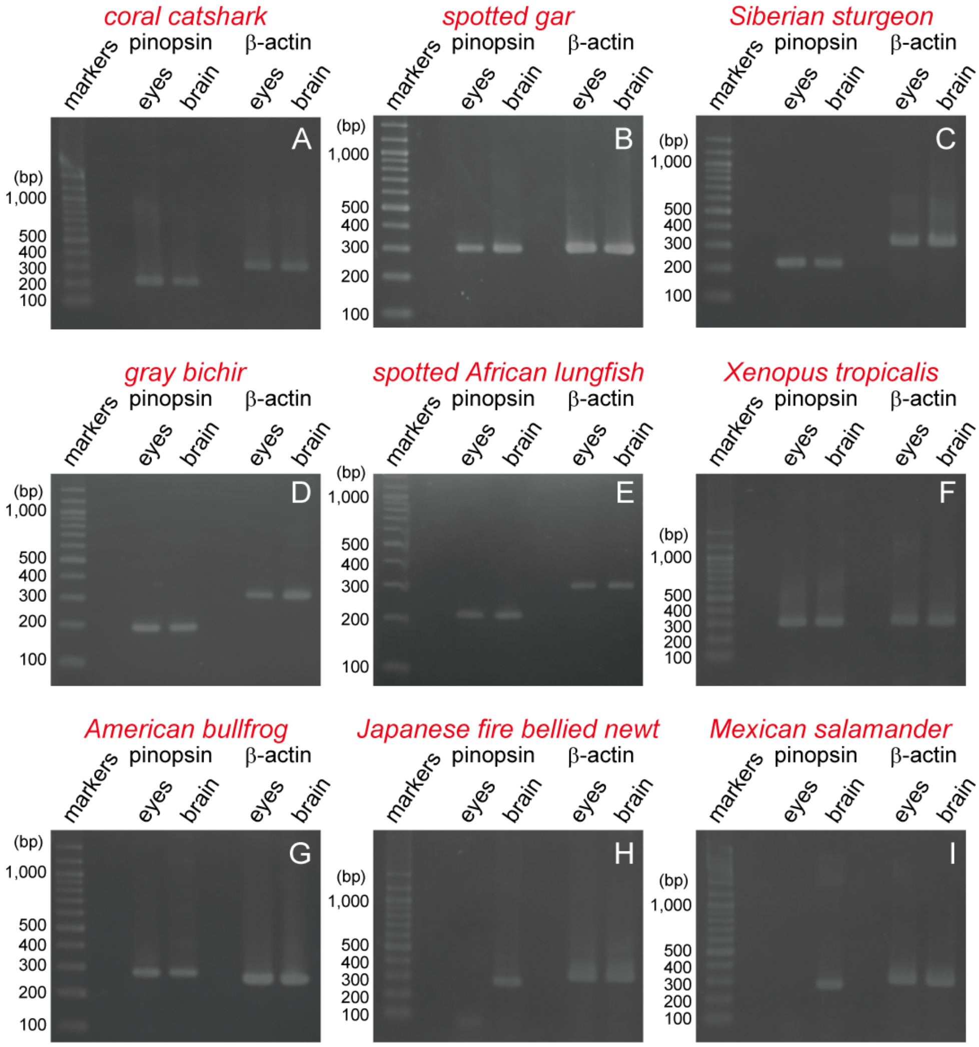

Supplementary Figure 2 RT-PCR analysis of pinopsin expression in eyes and brains
Pinopsin transcript was detected from both eyes and brain of coral catshark (A), spotted gar (B), Siberian sturgeon (C), gray bichir (D), spotted African lungfish (E), *X. tropicalis* (F) and American bullfrog (G). In contrast, the transcript was detected only from brain of Japanese fire bellied newt (H) and Mexican salamander (I). β-actin transcript was detected from all the samples as an internal standard. Sequences of PCR primers and amplified sizes of each PCR are shown in Supplementary Table 1.

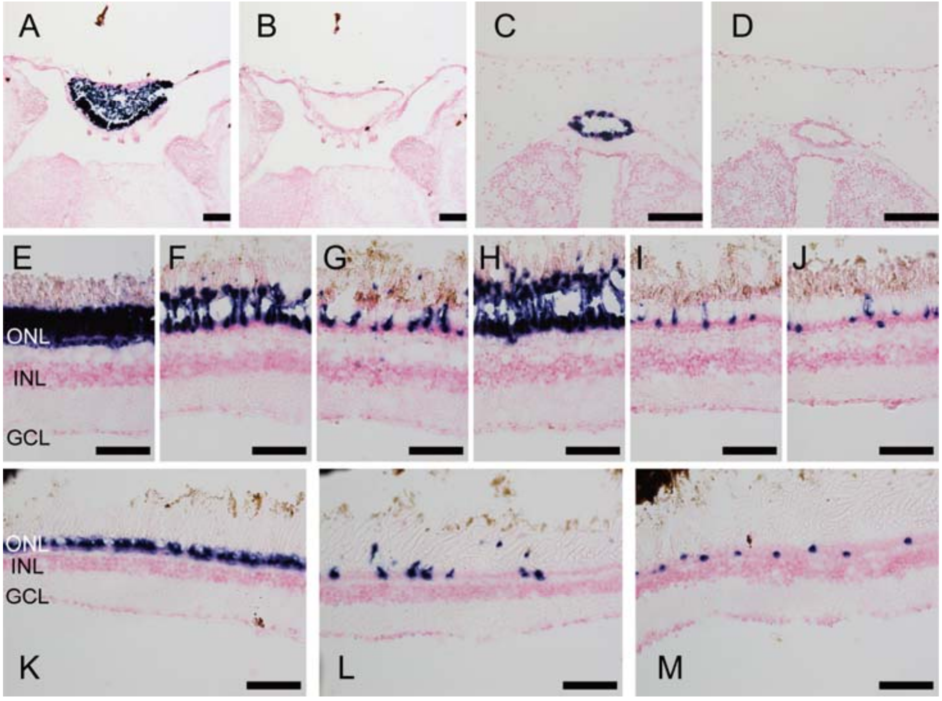

Supplementary Figure 3 Distribution of opsins in the retina and pineal gland of spotted gar and *X. tropicalis*

A-D, Pinopsin mRNA was detected in the pineal gland of spotted gar and *X. tropicalis* by *in situ* hybridization analysis. Brain frontal sections of spotted gar (A, B) and *X. tropicalis* (C, D) were hybridized with antisense (A, C) and sense (B, D) probes of pinopsin. E-J, Detection of spotted gar visual pigments in the retina by *in situ* hybridization analysis. Retinal sections were hybridized with antisense probes of rhodopsin (E), green-sensitive cone pigment (F), blue-sensitive cone pigment (G), red-sensitive cone pigment (H), UV-sensitive cone pigment1 (I) and UV-sensitive cone pigment2 (J). Q-S, Detection of *X. tropicalis* visual pigments in the retina by *in situ* hybridization analysis. Retinal sections were hybridized with antisense probes of red-sensitive cone pigment (K), blue-sensitive cone pigment (L) and violet-sensitive cone pigment (M). All the sections shown in this figure were counterstained with Nuclear Fast Red. Scale bar: A-D, 100 μm; E-M, 50μm.

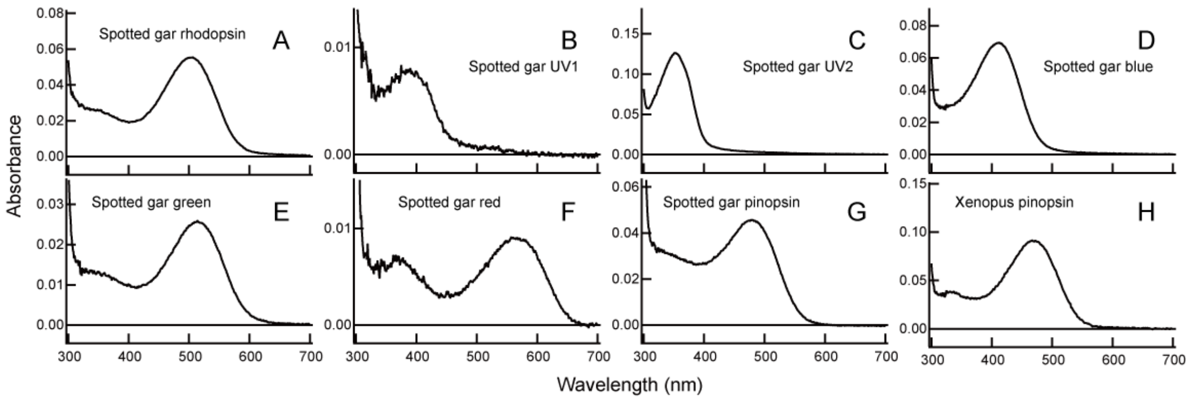

Supplementary Figure 4 Absorption spectra of spotted gar and *X. tropicalis* opsins

Absorption spectra of spotted gar rhodopsin (A), UV-sensitive cone pigment1 (B), UV-sensitive cone pigment2 (C), blue-sensitive cone pigment (D), green-sensitive cone pigment (E), red-sensitive cone pigment (F) and pinopsin (G) and *X. tropicalis* pinopsin (H) were measured after reconstitution with 11-*cis*-form of A1 retinal. All the spectra were recorded at 0 °C. λmax of opsins were 500 nm (spotted gar rhodopsin), 390 nm (spotted gar UV-sensitive cone pigment1), 353 nm (spotted gar UV-sensitive cone pigment2), 412 nm (spotted gar blue-sensitive cone pigment), 514 nm (spotted gar green-sensitive cone pigment), 560 nm (spotted gar red-sensitive cone pigment), 477 nm (spotted gar pinopsin) and 470 mm (*X. tropicalis* pinopsin). It should be noted that many fishes and amphibians also use A2 retinal as a chromophore to red-shift the λmax of the pigments[5]. The chromophore switch between A1 and A2 retinal during metamorphosis from tadpoles to adults has been observed in many frog species including Xenopus[6,7]. Although there is no information about the retinal chromophore in the spotted gar retina, the spotted gar genome contains the Cyp27c1 gene encoding the key enzyme to convert vitamin A1 into A2[8], which provides the possibility that spotted gar also uses A2 retinal in the retina.

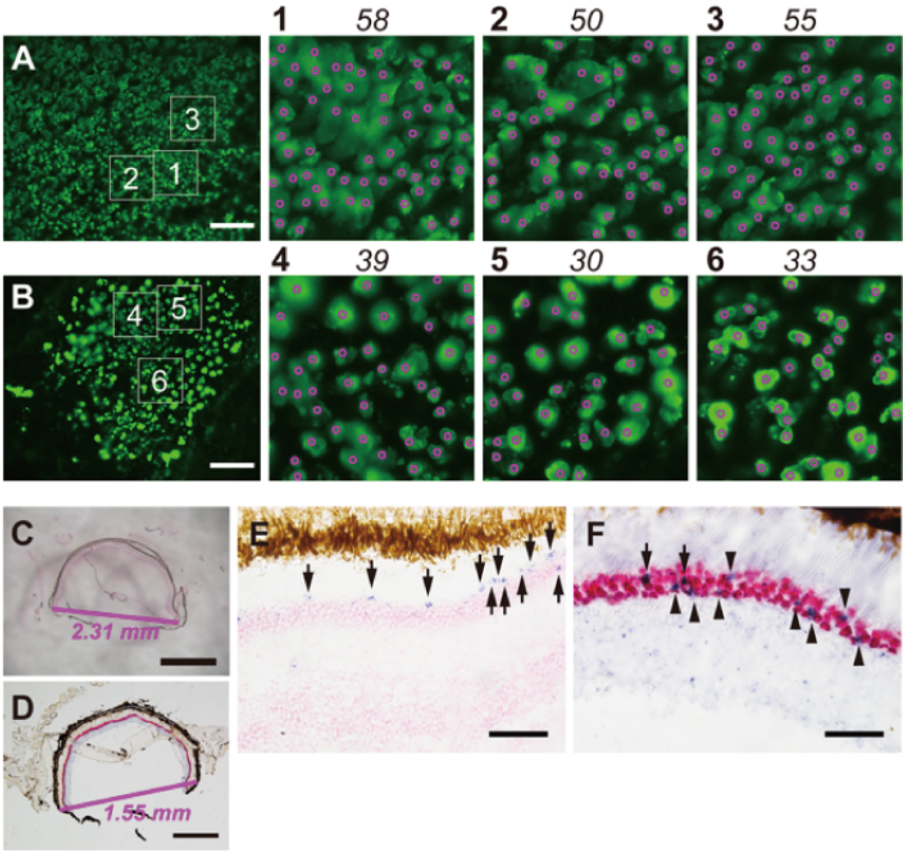

Supplementary Figure 5 Quantitative assessment of pinopsin-positive photoreceptor cell population

To approximate the ratio of pinopsin-expressing photoreceptor cells in the retina, density of rod photoreceptor cells, surface areas of retina, and the number of pinopsin-positive photoreceptor cells were estimated. A, B, Estimation of surface density of rod cells in the retina. Posterior part of the spotted gar (A) or *X. tropicalis* (B) retina was sectioned parallel to tangential plane and stained with anti-rhodopsin antibody. Three 50 µm square regions were chosen from each image to count the numbers of rod outer segments. Magenta circles show the positions of counted signals. The italic numbers indicate the numbers of counted signals in each panel. Averages were 54.3 ± 4.04 and 34.0 ± 4.58 cells in a 50 µm square for the spotted gar and *X. tropicalis* retina, respectively. Thus, the surface densities of rod cells in the spotted gar and *X. tropicalis* retina were estimated to be 2.17 and 1.36 × 10^4 mm^{-2}, respectively. This is comparable to the previous estimation in the *Xenopus laevis* retina[9]. C, D, Estimation of surface area of the retina. In the specimens used for counting the numbers of pinopsin-positive photoreceptor cells, the diameters of the spotted gar (C) and *X. tropicalis* (D) retina were 2.31 and 1.55 mm, respectively. Panel C image was taken by stereo microscope. Assuming that the retina has a hemisphere-like form, the surface areas of the spotted gar and *X. tropicalis* retina were estimated to be 8.38 and 3.77 mm^2. Thus, assuming a uniform surface density of rod cells, the numbers of rod cells were estimated to be 1.82 × 10^5 and 5.13 × 10^4 per spotted gar and *X. tropicalis* retina. Additionally, by referring to rod/cone ratio of *X. laevis*

retina (1.13:1)[9] and the presence of about 2.5 % of green rods in total rod cells[10], the numbers of cone cells in the *X. tropicalis* retina were estimated to be 4.66×10^4. E, F, Estimation of the numbers of pinopsin-expressing photoreceptor cells in the retina. The numbers of pinopsin-positive photoreceptor cells were estimated based on the results of *in situ* hybridization. E, Spotted gar retina was cryo-sectioned and placed consecutively on four separate slide glasses. Two slides were stained with digoxigenin-labelled antisense probe of pinopsin. The sections were counterstained with Nuclear Fast Red. Positive signals indicated by arrows were counted as pinopsin-expressing rod photoreceptor cells. F, *X. tropicalis* retina was cryo-sectioned and placed consecutively on eight separate slide glasses. Two slides were stained with fluorescein-labelled antisense probe of pinopsin and digoxigenin-labelled antisense probe of rhodopsin. After color development of NBT/BCIP for pinopsin and ImmPACT Vector Red for rhodopsin, blue precipitate signals overlapped (arrows) or non-overlapped (arrow heads) with red ones were counted as pinopsin-expressing cone and rod cells, respectively. Based on the results shown in (E) and (F), the numbers of pinopsin-positive cells per retina were estimated to be 732, 260, and 864 for spotted gar rod, *X. tropicalis* rod, and *X. tropicalis* cone, respectively. Therefore, the ratios of pinopsin-positive cells in spotted gar rods, *X. tropicalis* rods, and *X. tropicalis* cones were approximated to be 0.40, 0.51, and 1.9 %, respectively. Scale bar: A, B, E, F, 50 μm; C, 1 mm; D, 500 μm.

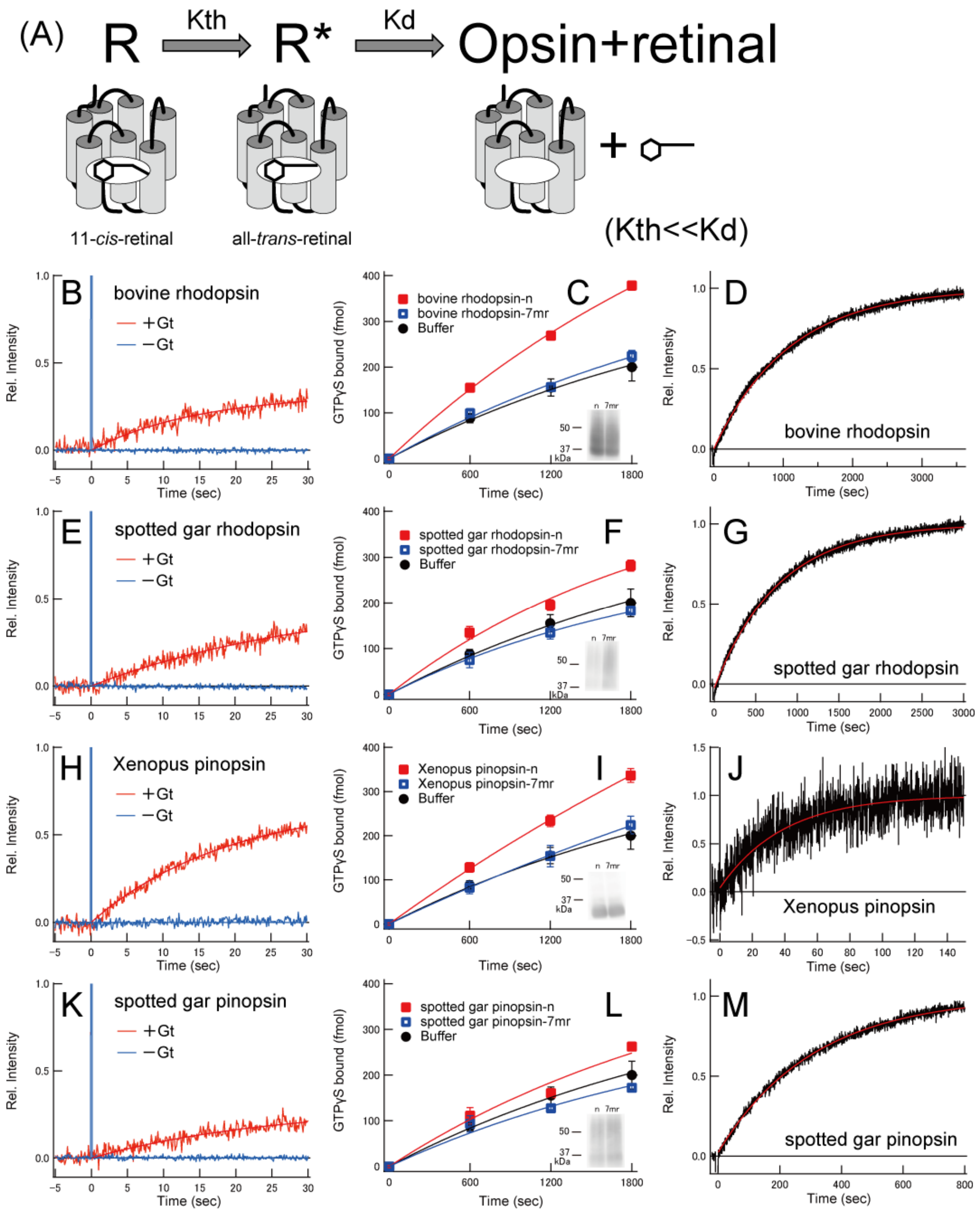

Supplementary Figure 6 Comparison of the thermal activation rate by the biochemical method.

A, Two-step reaction scheme of thermal activation and deactivation of visual pigments. R and R* indicate the inactive and active state of visual pigments, respectively. An opsin regenerated with normal 11-*cis*-retinal spontaneously converts to R* by thermal isomerization of retinal in the dark. After the first reaction, R* is degraded into opsin and all-*trans*-retinal. B, E, H, K, Measurements of the v_{light} by monitoring the change of intrinsic tryptophan fluorescence. The change of intrinsic tryptophan fluorescence after light irradiation with or without Gt are indicated by the red or blue line, respectively. Intensities

were normalized to the full fluorescence increase in the presence of aluminium fluoride. C, F, I, L, Measurements of the v_{dark} by a [^{35}S]GTPγS binding assay. Data from bovine rhodopsin-n, spotted gar rhodopsin-n, Xenopus pinopsin-n and spotted gar pinopsin-n are indicated by filled red squares. Data from bovine rhodopsin-7mr, spotted gar rhodopsin-7mr, Xenopus pinopsin-7mr and spotted gar pinopsin-7mr are indicated by open blue squares. The buffer control is indicated by filled black circles. Error bars represent the S.E.M of more than three independent measurements. The western blotting data were cropped and are shown in the inset (left lane: purified samples regenerated by both 11-*cis*-retinal and 7mr, right lane: purified samples regenerated by only 7mr). D, G, J, M, Measurements of the k_d by monitoring the change of intrinsic tryptophan fluorescence. The change of intrinsic tryptophan fluorescence after light irradiation without Gt are indicated by the black line.

X. tropicalis

TGTGATGCTAATCTTGTTAAGAAAAGTACAACATTTGTATATACTTACTTTGTTAATTAG
GTTGTATGACTTTGAGGCAGGTCACCTTCTAAAGAGCATCTGTTTCTGTTGATCTCTGTT
TAAGTAAAGAAAAAGAGAGTGGATTACAAAAACGCCAGAGTAAGAAAGCTTACTGTCTTT
TAAGAAAAAGATCCTCTTTGGAGCATTAAATCTCTTGGACTTTATAAACAGGGCTTTTAC
ACCAAGAGGAACACTTTTTTATTCATCTGCAAGCCTGAAAACAGTATTACAATG

spotted gar

CAGGGCACTAATTCCTCCTCCCTATATAAAGGGACCCCATGCCACGCTTGAGAAAGATTG
ATCGGCATCCCTCATTCTGCCTCTCCCTCCTGCACTCTCTCTGCATCTCACGATG

elephant shark

TCAAAACCTAATTATTGGAAAGAGCTGGAGGCCTGATCAGTGAAAGCCTTTTAGACTAAA
TCCCTCTGAAATATTTGATTAGCTGGAGTGGGTGTAATGAAAGATTAGGGCTCAGGTAAA
AAGGATCTATCGATGGAGGTGTAAAGCCTCGGTCCTTTATAATGGGACTTTACCACATGC
TGCTGCCCACATGCTGCCGACAATGACCAGGCTGCTCGATCTCCGCTGAGAGCATTAATC
CCAACAGTCCGTGAAGGCAATG

Supplementary Figure 7 The upstream sequences of pinopsin genes

ATG start codon, potential Crx/Otx-binding sites and TATA box in the upstream sequences of *X. tropicalis*, spotted gar and elephant shark pinopsin genes are colored in red, blue and green, respectively.

A

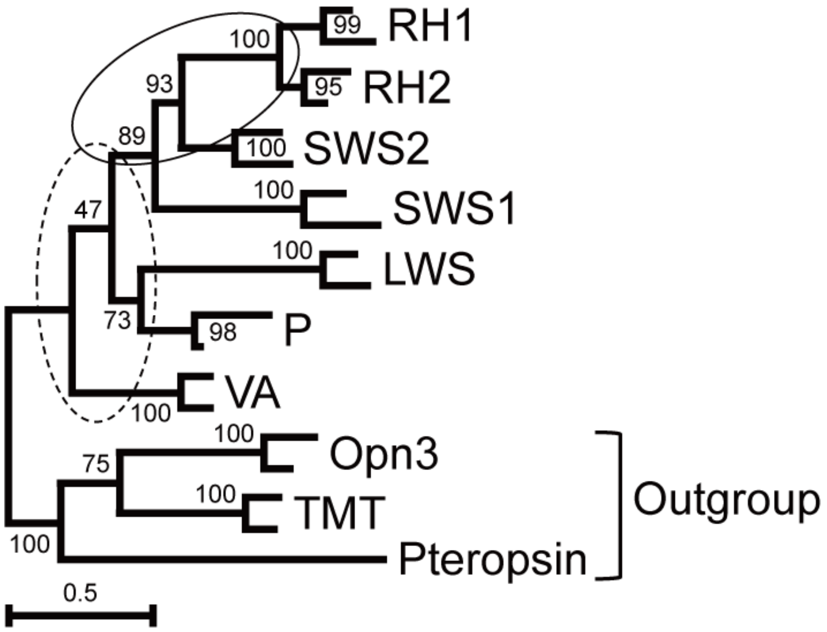

B

No.	tree topology	AU	KH	SH
1.	(((RH1,RH2),SWS2),SWS1,(LWS,P))	0.981	0.929	1.0
2.	(((RH1,RH2),(SWS2,SWS1),(LWS,P))	0.053	0.071	0.757
3.	(((RH1,RH2),SWS1),SWS2,(LWS,P))	0.053	0.071	0.757

C

No.	tree topology	AU	KH	SH
1.	(([[[RH1,RH2],SWS2],SWS1],(LWS,P)),VA,OUT)	0.802	0.664	0.942
2.	(([[[RH1,RH2],SWS2],SWS1],VA),(LWS,P),OUT)	0.580	0.336	0.809
3.	([[[RH1,RH2],SWS2],SWS1],((LWS,P),VA),OUT)	0.413	0.260	0.759
4.	((([[[RH1,RH2],SWS2],SWS1],LWS),P),VA,OUT)	0.327	0.158	0.429
5.	((([[[RH1,RH2],SWS2],SWS1],VA),P),LWS,OUT)	0.277	0.167	0.463
6.	([[[RH1,RH2],SWS2],SWS1],((P,VA), LWS),OUT)	0.248	0.147	0.301
7.	(([[[RH1,RH2],SWS2],SWS1],LWS),(P,VA),OUT)	0.201	0.122	0.203
8.	(([[[RH1,RH2],SWS2],SWS1],(P,VA)),LWS,OUT)	0.160	0.120	0.199
9.	((([[[RH1,RH2],SWS2],SWS1],VA),LWS),P,OUT)	0.124	0.140	0.421
10.	((([[[RH1,RH2],SWS2],SWS1],LWS),VA),P,OUT)	0.114	0.097	0.165
11.	((([[[RH1,RH2],SWS2],SWS1],P),VA),LWS,OUT)	0.092	0.078	0.151
12.	((([[[RH1,RH2],SWS2],SWS1],P),LWS),VA,OUT)	0.085	0.096	0.317
13.	(([[[RH1,RH2],SWS2],SWS1],P),(LWS,VA),OUT)	0.061	0.065	0.129
14.	([[[RH1,RH2],SWS2],SWS1],((LWS,VA),P),OUT)	0.030	0.080	0.186
15.	(([[[RH1,RH2],SWS2],SWS1],(LWS,VA)),P,OUT)	0.011	0.055	0.096

Supplementary Figure 8 Detailed analysis of phylogenetic relationship between pinopsin and visual pigments using multiple taxa from each opsin group.

A, Molecular phylogenetic tree of pinopsin and visual pigments inferred by maximum likelihood (ML) method. In order to examine the effect of single taxon (only chicken opsin gene) in each opsin group on ML analyses in Fig. 4, this analysis was performed by multiple

taxa (chicken and gar opsin genes) in each opsin group and three opsin groups (Opsin3, TMT opsin, and pteropsin) as an outgroup. The phylogenetic tree was constructed by RAxML[11] using JTT-tm model[2] and Yang's discrete gamma model[3] with an optimized shape parameter alpha of 0.89. An unambiguous sequence alignment of 249 amino acids in length was used for the tree inference. The numbers at each branch are bootstrap probabilities[4]. Partial tree topologies indicated by solid and broken ellipses were statistically tested in B and C, respectively. RH1, RH2, SWS1, SWS2, LWS, P, VA, Opn3, and TMT denote rhodopsin, green-sensitive opsin, violet-sensitive opsin, blue-sensitive opsin, red-sensitive opsin, pinopsin, vertebrate ancient opsin, Opsin3, and TMT opsin, respectively, and Pteropsin denotes western honey bee (*Apis mellifera*) pteropsin. For each two external tree branches of the nine opsin groups, RH1, RH2, SWS1, SWS2, LWS, P, VA, Opn3, and TMT, the upper one corresponds to a chicken gene and the lower one does to a gar gene. Accession numbers of chicken, spotted gar, and honey bee amino acid sequence data are the same as those in Supplementary Figure 1. B, ML and other two tree topologies of six opsin groups not statistically rejected by AU test. The ML tree of six opsin groups was inferred by the same method and models (alpha = 0.73) of A. A highly reliable sequence alignment of 290 amino acids in length was used for the tree construction. Out of all the 105 possible tree topologies for six opsin groups, RH1, RH2, SWS1, SWS2, LWS, and P, the ML (tree number 1) and only two (tree number 2 and 3) topologies were not rejected by AU test[12] at the five percent statistical significance level. The values of Kishino-Hasegawa (KH)[13] and Shimodaira-Hasegawa (SH)[14] statistical tests for these tree topologies are also represented in the list. In these three ML statistical tests for the 105 tree topologies, chicken and spotted gar genes of each opsin group were fixed in advance to make a cluster in these phylogenetic trees. C, ML and the other 14 tree topologies of ten opsin subgroups. Partial tree topology denoted by [[[RH1, RH2], SWS2], SWS1] was fixed in this analysis because the topology was contained in the ML tree of A and strongly supported by the statistical test of B. 'OUT' corresponds to the partial tree topology of the outgroup genes, [[Opn3, TMT], Pteropsin], which was also fixed in this analysis. Out of all the 15 possible tree topologies for five gene groups, [[[RH1, RH2], SWS2], SWS1], LWS, P, VA, and OUT, the ML (tree number 1) and other 12 (tree numbers 2 to 13) topologies were not rejected by AU test at the five percent level of significance. The values of KH and SH tests are also shown in the list. In these three ML statistical tests for the 15 tree topologies, chicken and spotted gar genes of each opsin group were fixed to make a cluster in these phylogenetic trees.

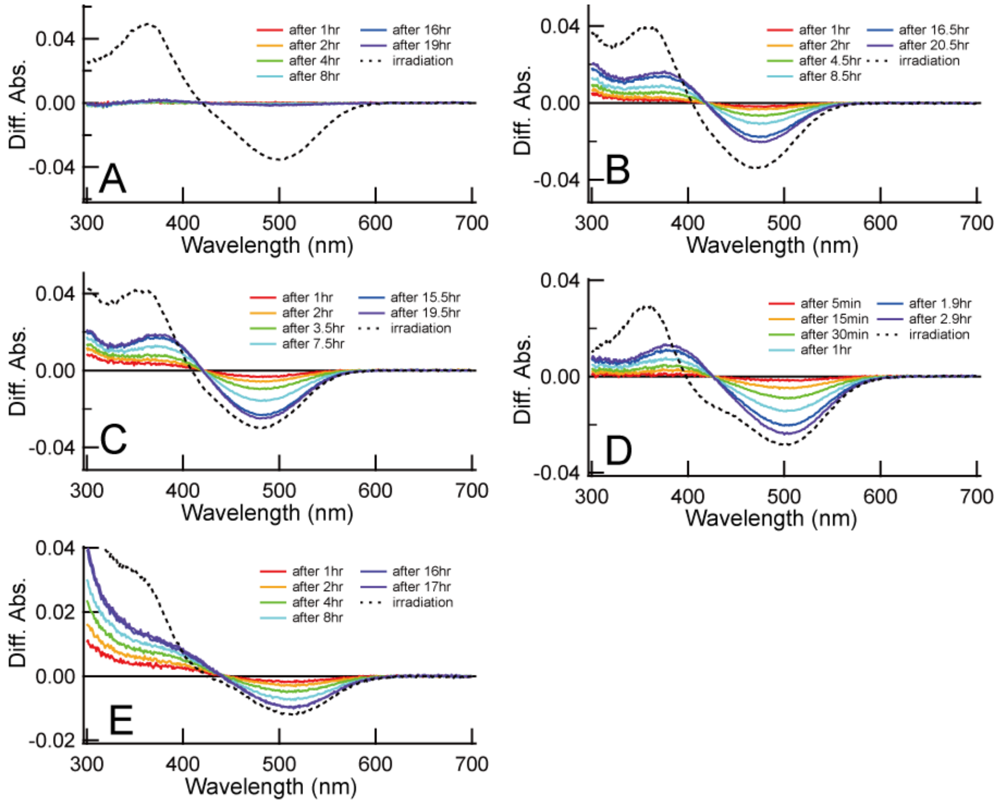

Supplementary Figure 9 Comparison of the spectral changes of rhodopsin, pinopsin and cone pigments during incubation at 37 °C. Difference spectra of bovine rhodopsin (A), Xenopus pinopsin (B), spotted gar pinopsin (C), chicken green-sensitive cone pigment (D) and mouse green-sensitive cone pigment (E) were obtained by subtracting the spectrum before incubation at 37 °C from those measured after incubation and irradiation shown in Fig. 5.

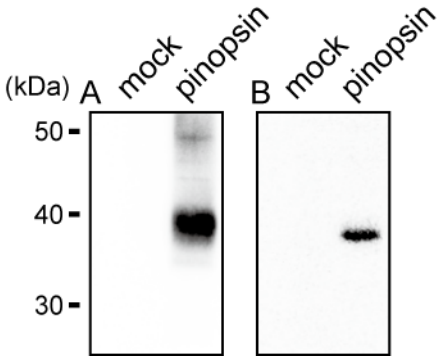

Supplementary Figure 10 Characterization of anti-pinopsin antibodies by Western blotting

A clear signal was detected in pinopsin-transfected cells but not in mock-transfected cells by anti-spotted gar pinopsin antibody (A) or anti-Xenopus pinopsin antibody (B).

Supplementary Table 1 List of primers used for RT-PCR analysis and sizes of amplified RT-PCR products in Fig. 1 and Supplementary Figure 2

clone	primers (forward / reverse)	amplified size
coral catshark pinopsin	CCCTGAGTCTGATCATCTTC (forward)	216bp
	AGGTGGGGTGAATCACGATG (reverse)	
spotted gar pinopsin	GAACGCTACATTGTCATCTG (forward)	308bp
	TTTTGTTGTGCTGCCACAGC (reverse)	
Siberian sturgeon pinopsin	TGGGCAAAGCTGTGTGCAAG (forward)	220bp
	TAGCTGCTCCAGCCAAAGAG (forward)	
gray bichir pinopsin	TGGGAACCTTCTGGTTACAC (forward)	197bp
	TGGAGGCTAGAGTTGGATTG (reverse)	
spotted African lungfish pinopsin	GGTAGCACAATCAGCTTCTC (forward)	204bp
	ACAGCCTATTACTGCATGCC (reverse)	
X. tropicalis pinopsin	AGCAAACTTGCTGGTCACAA (forward)	312bp
	GTCCTCAAACCTTCTGGAAC (reverse)	
American bullfrog pinopsin	TCTTGGCAAAGCGTTGTGTG (forward)	281bp
	TGTTTGTACCTCCGGAGTAC (reverse)	
Japanese fire bellied newt pinopsin	GGTACATTGTGATCTGTAAG (forward)	278bp
	AGTCATTAGTAGGCTGGTGT (reverse)	
Mexican salamander pinopsin	CTGTGATGGGCTGTGCATTC (forward)	297bp
	CCATCCTTGTCACCTCCTTC (reverse)	
coral catshark β-actin	CTGAAGAACATCCAGTCCTG (forward)	330bp
	TTTCTCTCTCAGCTGTGGTG (reverse)	
spotted gar β-actin	CTGAGGAGCACCCTGTTCTG (forward)	324bp
	TTTCGGCTGTGGTGGTGAAG (reverse)	
Siberian sturgeon β-actin	ATGTACGTTGCCATCCAGGC (forward)	325bp
	AGCTCTTCTCCAGGGAGGAA (reverse)	
gray bichir β-actin	CTGAGGAACACCCAGTGTTG (forward)	323bp
	TTCAGCTGTGGTTGTGAAGC (reverse)	
spotted African lungfish β-actin	TACAATGAGCTCCGTGTTGC (forward)	296bp
	AGGTAGTCTGTCAAGTCACG (reverse)	
X. tropicalis β-actin	CAGAGGAACACCCAGTGCTG (forward)	330bp
	TTTCTCTTTCAGCTGTGGTG (reverse)	
American bullfrog β-actin	ACTACCTCATGAAGATCCTG (forward)	269bp
	TCGTGAATGCCGCATGATTC (reverse)	
Japanese fire bellied newt β-actin	GTATGTGGCTATCCAGGCTG (forward)	322bp
	GCTCTTCTCCAGAGAAGATG (reverse)	
Mexican salamander β-actin	TGTTGCCATTCAAGCTGTGC (forward)	317bp
	TTTCTCCAGCGAAGACGAAG (reverse)	

Supplementary references

1. Saitou, N. & Nei, M. The neighbor-joining method: a new method for reconstructing phylogenetic trees. *Mol. Biol. Evol.* **4,** 406–425 (1987).
2. Jones, D. T., Taylor, W. R. & Thornton, J. M. The rapid generation of mutation data matrices from protein sequences. *Comput. Appl. Biosci. CABIOS* **8,** 275–282 (1992).
3. Yang, Z. Maximum likelihood phylogenetic estimation from DNA sequences with variable rates over sites: approximate methods. *J. Mol. Evol.* **39,** 306–314 (1994).
4. Felsenstein, J. Confidence limits on phylogenies : An approach using the bootstrap. *Evol. Int. J. Org. Evol.* **39,** 783–791 (1985).
5. Dartnall, H. J. & Lythgoe, J. N. The spectral clustering of visual pigments. *Vision Res.* **5,** 81–100 (1965).
6. Wald, G. The metamorphosis of visual system in Amphibia. *Biol. Bull.* **91,** 239 (1946).
7. Crescitelli, F. The visual pigment system of *Xenopus laevis* Tadpoles and adults. *Vision Res.* **13,** 855–865 (1973).
8. Enright, J. M. *et al.* Cyp27c1 red-shifts the spectral sensitivity of photoreceptors by converting vitamin A1 into A2. *Curr. Biol. CB* **25,** 3048–3057 (2015).
9. Wilhelm, M. & Gábriel, R. Functional anatomy of the photoreceptor and second-order cell mosaics in the retina of *Xenopus laevis*. *Cell Tissue Res.* **297,** 35–46 (1999).
10. Röhlich, P. & Szél, A. Photoreceptor cells in the Xenopus retina. *Microsc. Res. Tech.* **50,** 327–337 (2000).
11. Stamatakis, A. RAxML version 8: a tool for phylogenetic analysis and post-analysis of large phylogenies. *Bioinforma. Oxf. Engl.* **30,** 1312–1313 (2014).
12. Shimodaira, H. An approximately unbiased test of phylogenetic tree selection. *Syst. Biol.* **51,** 492–508 (2002).
13. Kishino, H. & Hasegawa, M. Evaluation of the maximum likelihood estimate of the evolutionary tree topologies from DNA sequence data, and the branching order in hominoidea. *J. Mol. Evol.* **29,** 170–179 (1989).
14. Shimodaira, H. & Hasegawa, M. Multiple comparisons of log-likelihoods with applications to phylogenetic inference. *Mol. Biol. Evol.* **16,** 1114–1114 (1999).
